# Microbiome-derived reactivation of mycophenolate explains variations in enterohepatic recirculation in kidney transplant recipients

**DOI:** 10.1186/s40168-025-02142-6

**Published:** 2025-07-24

**Authors:** Ole Martin Drevland, Eric J. de Muinck, Pål Trosvik, Marta Hammerstad, Kine Eide Kvitne, Karsten Midtvedt, Anders Åsberg, Ida Robertsen

**Affiliations:** 1 Section for Pharmacologyand, Pharmaceutical Biosciences, Department of Pharmacy , University of Oslo, Oslo, Norway; 2 Centre for Ecological and Evolutionary Synthesis, Department of Biosciences, University of Oslo, Oslo, Norway; 3 Section for Biochemistry and Molecular Biology, Department of Biosciences, University of Oslo, Oslo, Norway; 4 Skaggs School of Pharmacy and Pharmaceutical Sciences, University of California San Diego, La Jolla, CA USA; 5 Department of Transplantation Medicine, Oslo University Hospital, Oslo, Norway

**Keywords:** Mycophenolate, β-glucuronidase, Gut microbiome, Enterohepatic recirculation, Reactivation, *Faecalibacterium prausnitzii*

## Abstract

**Background:**

The pivotal role of microbes in drug metabolism is increasingly recognized, as variation in the gut microbiome composition between individuals has been shown to impact systemic drug exposure, efficacy and toxicity. Mycophenolate mofetil (MMF) is a cornerstone in immunosuppressive therapy following solid organ transplantation. However, dosing and tolerance are challenged by significant pharmacokinetic variability among patients, largely due to variable degrees of enterohepatic recirculation of mycophenolic acid (MPA), the active moiety of MMF. It is hypothesized that the variability in MPA recirculation is driven by gut microbiome-derived β-glucuronidase (β-GUS) mediated cleavage of MPA-glucuronide (MPAG) excreted in the bile. Here, we investigated the bidirectional interaction between MPA and the gut microbiome in kidney transplant recipients, using a combination of in vivo and in vitro data.

**Results:**

We compared the fecal microbiomes of kidney transplant recipients (*n* = 21) both pre- and post-transplantation to healthy individuals (*n* = 15) using shotgun metagenomic sequencing. We also determined the individual microbiome-derived reactivation rate of MPAG to MPA and show a strong positive correlation between this reactivation rate and the degree of MPA enterohepatic recirculation in vivo. Through metagenomic analysis, the reactivation rate of MPA was linked to specific gut microbial species. In particular, specific β-GUS gene variants associated with *Faecalibacterium prausnitzii* showed a strong impact on the conversion of MPAG to MPA. Furthermore, our study confirmed a significant shift in microbial composition post-transplantation and revealed notable fluctuations in species such as *F. prausnitzii* and *Akkermansia muciniphila* across different time points after transplantation. Lastly, we provide evidence that the microbiome-derived reactivation rate of MPA is linked to specific beta-glucuronidase alleles.

**Conclusions:**

We highlight for the first time that the ex vivo determined reactivation rate of MPA explains the variation of enterohepatic recirculation, emphasizing the important role of *F. prausnitzii* in this process. More broadly, our findings suggest that the gut microbiome significantly influences the degree of enterohepatic recirculation of MPA, providing valuable insights that could be relevant for optimizing individualized immunosuppressive drug dosing in transplant patients.

Video Abstract

**Supplementary Information:**

The online version contains supplementary material available at 10.1186/s40168-025-02142-6.

## Background

The field of pharmacomicrobiomics, investigating how variations in the microbiome impact drug disposition, action, and toxicity, is gaining tremendous interest [1]. Mechanistically, the gut microbiome can influence drug disposition both directly through chemical transformation and bioaccumulation of drugs in the gastrointestinal (GI) tract, or indirectly by interacting with host enzymes and transporters [2, 3]. Numerous structurally diverse drugs can be metabolized by gut microbes through a wide range of reactions including reduction, hydrolysis, deamination, phosphorolysis, and decarboxylation [4, 5]. Such modifications can have profound effects on drug exposure and partly explain the considerable interindividual variability in drug response [1]. Conversely, drug exposure can cause shifts in gut microbiome composition, altering the metabolic potential of the microbiome [6]. Consequently, understanding the bidirectional relationship between drugs and the gut microbiome is crucial for optimizing therapeutic strategies and minimizing adverse effects.

Following solid organ transplantation, lifelong immunosuppressive therapy is required to prevent organ rejection. Previous studies have revealed that solid organ transplantation induces changes in the gut microbiome, which have been linked to increased morbidity and mortality in these patients [7, 8]. This transplant-induced dysbiosis is characterized by reduced microbial diversity, a decrease in important metabolic pathways and an increased abundance of pathogenic microbial species [7, 9]. Notably, immunosuppressive drugs have been identified as the single most important factor underlying the observed post-transplant gut dysbiosis [8].

Mycophenolate mofetil (MMF), a prodrug of mycophenolic acid (MPA), is a cornerstone of modern immunosuppressive therapy in solid organ transplantation [10, 11]. MPA is inactivated predominantly in the liver by uridine diphosphate-glucuronosyltransferases (UGTs), producing the main metabolite 7-O-MPA-glucuronide (MPAG), which is excreted to the GI tract via the biliary ducts [12, 13]. In the intestine, bacterial β-glucuronidase (β-GUS) enzymes cleave off the glucuronic acid to reactivate MPA which then is available for re-absorption into the systemic circulation. The resulting glucuronic acid is available as a carbon source for bacterial metabolism [14]. This enterohepatic recirculation contributes up to 40% of the total MPA systemic exposure, affecting both degree of immunosuppression and potential toxicity [15, 16]. Studies have shown that dose-normalized systemic exposure of MPA can vary more than tenfold [17, 18], reflecting considerable interindividual variability in pharmacokinetics, complicating MMF dosing. A large part of the variability remains unexplained, and the gut microbiome may play a significant role in the differences observed in systemic exposure of MPA. Consequently, understanding the interactions between MPA and the gut microbiome could help develop better individualized dosing strategies in solid organ recipients.

The microbiome-encoded β-GUS enzymes play important roles in human health [19–24], with numerous unique gut microbiome-derived β-GUS enzymes identified [25]. Among these, flavin mononucleotide (FMN) binding β-GUS enzymes have been shown to drive the reactivation of MPA from MPAG [26]. Despite this, the specific microbial species responsible for this reactivation and the correlation to in vivo pharmacokinetic data remain unclear. Here, we investigated the bidirectional interaction between MPA and the gut microbiome in kidney transplant recipients. We hypothesized that the degree of MPA enterohepatic recirculation would be linked to the microbiome’s capacity to reactivate MPA, reflecting the gut bacterial community’s influence on drug metabolism. Using shotgun metagenomic sequencing, we compared the microbiome composition of kidney transplant recipients pre- and post-transplantation with healthy individuals to identify microbial factors associated with MPA reactivation. Additionally, we examined the presence of specific β-GUS alleles to understand their potential role in the substrate-specific conversion of MPAG back to MPA. Together, these analyses provide a novel understanding on how the gut microbiome may contribute to the variability in MPA exposure, paving the way for more personalized immunosuppressive therapy in transplant recipients.

## Results

### Study population and fecal sampling

As outlined in Supplementary Fig. S1a, fecal samples were collected from 22 kidney transplant recipients. The kidney transplant recipients had ages ranging from 28 to 77 years (median 55 years), median BMI of 27 kg/m^2^ (range 20 to 35 kg/m^2^) and 73% were men. Throughout the study period, the MMF dose was maintained at 750 mg twice daily for all patients. Further details regarding the demographic data and patient characteristics of the kidney transplant recipients at the time of transplantation can be found in Supplementary Table S1. None of the transplant recipients received primary antibiotic prophylaxis but they all received *Pneumocystis jirovecii* pneumonia prophylaxis with sulfamethoxazole/trimethoprim (400/80 mg/day) for 6 months after transplantation. Additionally, one patient was on a 1-week regimen with pivmecillinam (600 mg/day) 18 days after transplantation. A list of all drug classes used by the patients can be found in Supplementary Table S2. Twenty-two transplanted patients provided fecal samples prior to transplantation, 21 1 week after transplantation, 11 between 3 and 6 weeks after transplantation, and 8 1 year after transplantation.

Fecal samples were collected from 15 healthy individuals (Supplementary Fig. S1b). The ages ranged from 21 to 52 years (median 24 years), median BMI of 23 kg/m^2^ (range 18 to 29 kg/m^2^), and 60% were women. Among these individuals, 7 refrained from using any medication, while 8 individuals were using hormonal contraception.

The mean time from fecal sampling to – 80 °C storage was 7.7 ± 2.4 h for the kidney transplant recipients and 13.9 ± 1.0 h for the healthy individuals. No statistically significant differences in time from sampling to – 80 °C storage was observed across the visits for the patients. Additionally, the mean time between fecal sampling and start of the pharmacokinetic investigation day was 8.2 ± 4.5 h for the kidney transplant recipients who underwent these investigations.

### Microbiome-derived reactivation rate of MPA is strongly correlated with the degree of enterohepatic recirculation of MPA in vivo

**Fig. 1:**
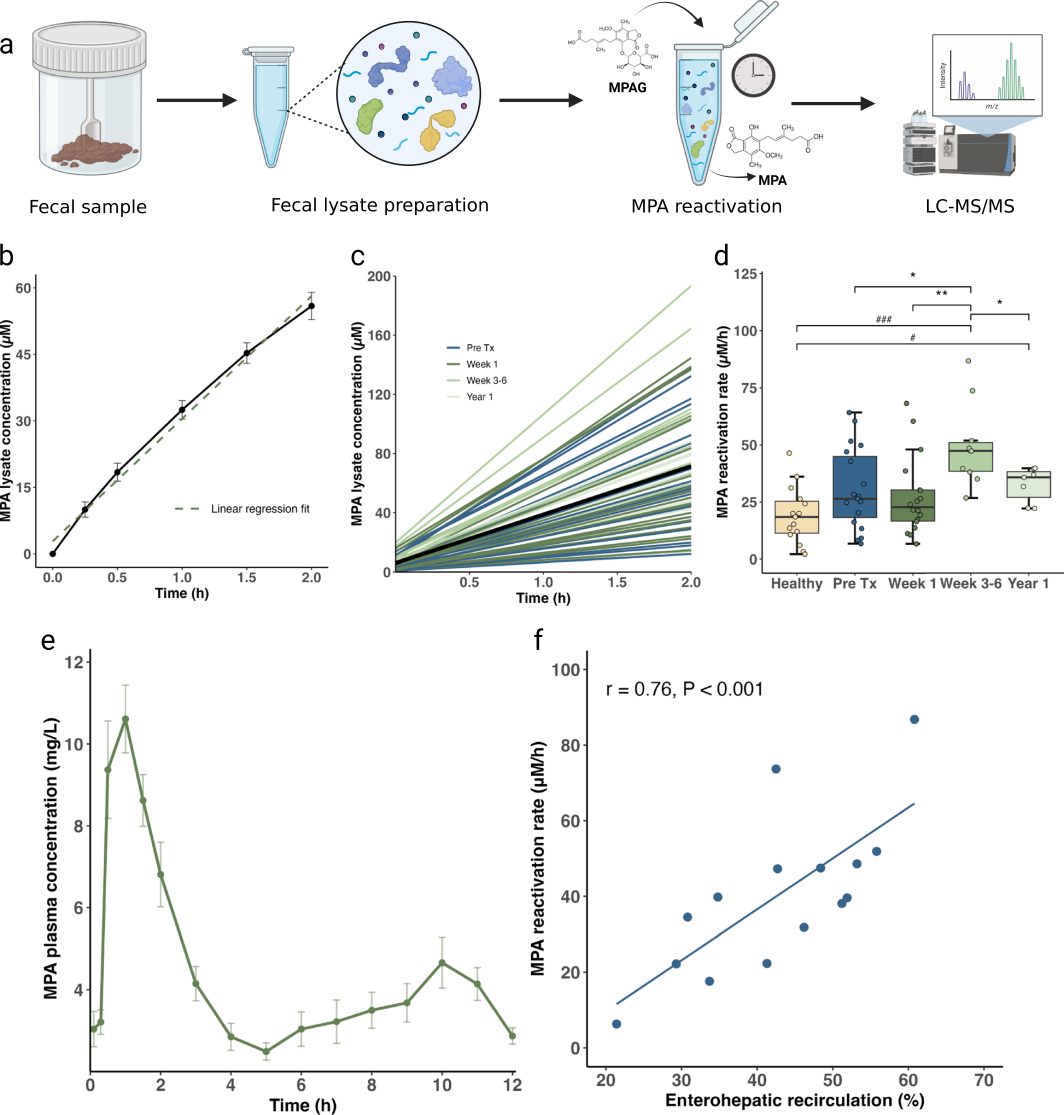
Microbiome-derived reactivation of MPA ex vivo explains variations in enterohepatic recirculation of MPA in vivo.** a** Overview of the experimental method used to investigate the microbiome-derived reactivation of mycophenolic acid (MPA) from 7-O-mycophenolic acid glucuronide (MPAG) in human fecal lysates. High molecular weight proteins were purified from fecal samples and the resulting fecal lysates were exposed to MPAG. MPA plasma concentrations were measured using UPLC-MS/MS. **b** Visualization of the reactivation of MPA from MPAG in one of the kidney transplant recipients. MPA concentrations as a function of time were fitted to a linear regression model (green dashed line), with the slope representing the reactivation rate. **c** Linear regressions fits for the individual kidney transplant patients and overall mean (black line). Fecal samples from all visits (*n* = 62) are represented. **d** Grouped comparison of MPA reactivation rates (μM/h) from healthy individuals (*n* = 15) with kidney transplant recipient at different time points; pre-transplantation (Pre-Tx, *n* = 22), one week after transplantation (week 1, *n* = 21), between 3 to 6 weeks after transplantation, (week 3–6, *n* = 11) and one year after transplantation (year 1, *n* = 8). **e** Mean (± SEM) concentration–time profile of MPA showing the secondary absorption peak caused by enterohepatic recirculation (*n* = 15). **f** Correlation between MPA reactivation rates (μM/h) and the degree of enterohepatic recirculation (%) estimated using the Spearman’s correlation test (*n* = 15). */^#^p < 0.05; **/^##^*p* < 0.01; ***/^###^*p* < 0.001. Comparisons between kidney transplant recipients are indicated by an asterisk (*), while comparisons between kidney transplant recipients and healthy individuals are indicated by a hash symbol (^#^)

To investigate the gut microbiome-derived reactivation of MPA from MPAG in kidney transplant recipients and healthy individuals, we processed the fecal samples as outlined in Fig. 1a and determined the reactivation rate of individual gut microbiomes by using a linear regression model as shown in Fig. 1b (see also “Methods” section). We analyzed a total of 62 and 50 samples from kidney transplant recipients and healthy individuals, respectively. MPA reactivation rates varied between 6 and 86 µM/h in the samples from kidney transplant recipients (Fig. 1c) and between 2 and 46 µM/h in the samples from healthy individuals, demonstrating a 14- and 23-fold difference, respectively. The mean MPA reactivation rates over time in kidney transplant recipients and in healthy individuals are illustrated in Fig. 1d. Notably, the mean reactivation rate was significantly lower both pre-transplantation (30.3 ± 17.6 µM/h) and at the 1-week visit (27.2 ± 16.9 µM/h) compared to 3–6 weeks after transplantation (49.5 ± 18.1 µM/h) (Wilcoxon signed rank test; *p* = 0.014 and *p* < 0.001, respectively; Fig. 1d). Additionally, the ability to reactivate MPA from MPAG was lower 1 year after transplantation (32.5 ± 7.6 µM/h) compared to 3 to 6 weeks after transplantation (*p* = 0.031). There was no statistically significant difference in MPA reactivation rates between healthy individuals (19.2 ± 12.3 µM/h) and kidney transplant recipients pre-transplantation (*p* = 0.072) and 1 week after transplantation (*p* = 0.16). However, MPA reactivation rates were significantly higher in kidney transplant recipients 3 to 6 weeks (*p* < 0.001) and 1 year post-transplantation (*p* = 0.014) compared to healthy individuals. To validate the method and account for inherent variation, we included control samples of Anaerobic Cultivated Human Intestinal Microbiota (ACHIM), a standardized, feces-based culture. The ACHIM control samples exhibited minimal experimental deviation, with a coefficient of variation < 7% (Supplementary Table S3).

Further, we investigated if individual MPA reactivation rates could explain the degree of enterohepatic recirculation. In 10 kidney transplant recipients, we performed 12-h pharmacokinetic investigations of MMF, including 16 blood samples, at 3 to 6 weeks post-transplantation. Five of these patients repeated the investigation at 1 year, resulting in a total of 15 pharmacokinetic profiles of MPA. From these profiles, we determined the proportion of total AUC_0–12_ that could be attributed to enterohepatic recirculation in vivo. The mean (± SEM) concentration–time profile of MPA show a second peak around 6 to 10 h after dosing due to enterohepatic recirculation (Fig. 1e). This enterohepatic recirculation is highly variable both between and within individuals. Individual plasma concentration–time profiles of MPA are illustrated in Supplementary Fig. S2. Importantly, we found a strong correlation between the MPA reactivation rates and the degree of enterohepatic recirculation in kidney transplant recipients (Spearman’s correlation test; *r* = 0.76, *p* < 0.001; Fig. 1f). However, there was no correlation between the MPA reactivation rates and either AUC_0–12_ or trough concentrations (C_12_) of MPA (Spearman’s correlation test; *r* = 0.30, *p* = 0.29 and *r* = 0.30, *p* = 0.30, respectively; Supplementary Fig. S3).

We also quantified general β-GUS activity using a commercial assay kit to evaluate whether this approach could be as informative as our novel MPA reactivation rate metric in explaining variation in the degree of enterohepatic recirculation. Moderate correlations was observed between general β-GUS activity and both the degree of enterohepatic recirculation (Spearman’s correlation test; *r* = 0.43, *p* = 0.107) and MPA reactivation rates (*r* = 0.28, *p* = 0.315) (Supplementary Fig. S4). This indicates that a commercial kit measuring general β-GUS activity may be less sensitive in assessing specific reactivation reactions.

### Taxonomic diversity in fecal metagenomes

**Fig. 2:**
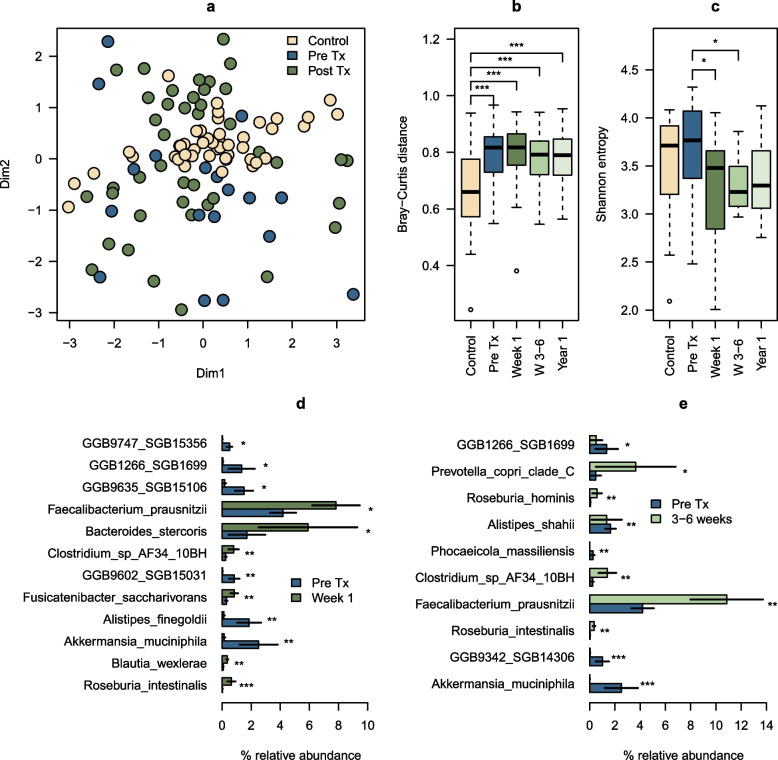
Composition and diversity of the gut microbiome in healthy individuals and kidney transplant recipients. **a** Nonparametric multidimensional scaling (nMDS) plot showing differences in microbial community compositions among samples based on Bray–Curtis dissimilarities. Each point represents either a fecal sample from a healthy individual (beige) or a kidney transplant recipient (blue = pre-transplantation, green = post-transplantation). Kidney transplant recipients have an altered GI microbiome compared to healthy individuals (*R*.^2^ = 0.06, *p* < 0.001). The final stress value of the nMDS model was 24.2, indicating a relatively poor fit. **b** Comparison of Bray–Curtis distances between healthy individuals and kidney transplant recipients. The mean distance among the kidney transplant recipients (0.79 (± 0.10 SD) was higher than that of healthy individuals 0.64 (± 0.14 SD) regardless of the time elapsed since transplantation (*p* < 0.001). **c** Boxplot comparing the Shannon Diversity index in healthy individuals (*n* = 15) with kidney transplant recipients pre-transplantation (Pre-Tx, *n* = 22), 1 week post-transplantation (week 1, *n* = 21), 3 to 6 weeks post-transplantation (week 3–6, *n* = 11) and 1 year post-transplantation (year 1, *n* = 8). **d**,** e** Barplots illustrating statistically significant differences in taxa relative abundances between fecal samples obtained pre-transplantation, 1 week (**d**) and 3 to 6 weeks post-transplantation (**e**). The analysis was conducted using DESeq2. (Differential Expression Sequencing) and was restricted to taxa that were observed with relative abundances of at least 0.01% in at least half of the samples for a given test combination. *p < 0.05; **p < 0.01; ***p < 0.001. Dim1, dimension 1; Dim2, dimension 2

The shotgun metagenomic sequencing of DNA extracted from fecal samples from both the kidney transplant patients and healthy individuals, generated a mean library size of 41,214,638 reads (range: 2,966,282–116,921,150) after quality trimming and filtering of host-derived reads. The strongest structuring factor for β-diversity was the individual provenance of the samples (*R*^2^ = 0.76, *p* < 0.001; PERMANOVA), while the altered microbial composition in kidney transplant recipients compared to healthy individuals was also highly significant (*R*^2^ = 0.06, *p* < 0.001; Fig. 2a). We did not find significant differences in β-diversity pre-transplantation vs. 1 week, 3 to 6 weeks or 1 year post-transplantation. Furthermore, there was a significant association between β-diversity and the MPA reactivation rates (*R*^2^ = 0.02, *p* = 0.002; PERMANOVA). The mean Bray–Curtis distance in the kidney transplant recipients was significantly higher than that of healthy individuals in pre-transplant samples as well as at all post-transplant time points (*p* < 0.001, unpaired *t*-test; Fig. 2b), indicating a greater variability and less uniformity in the microbiome composition of kidney transplant recipients compared to healthy individuals. The fecal samples from kidney transplant recipients showed similar α-diversity to those from healthy individuals, as measured by the Shannon Diversity Index (Fig. 2c). However, the mean Shannon Diversity index was significantly decreased 1 week (3.24 ± 0.58 SD, *p* = 0.016) and 3 to 6 weeks post-transplantation (3.30 ± 0.31 SD, *p* = 0.017), compared to pre-transplantation levels (3.69 ± 0.44 SD). In summary, these results suggest that while individual provenance remains the primary factor driving microbial community structure, kidney transplantation significantly alters microbial composition without impacting overall α-diversity. Lastly, there was a significant trend for the species level composition of the gut microbiome of transplant patients to become more similar to the healthy individuals over time (Wilcoxon signed rank test; all *p* < 0.05; Supplementary Fig. S5).

### Differences in microbiome composition pre –and post-transplantation

The initial findings prompted us to investigate the specific difference in microbiome composition before and after kidney transplantation. Using DESeq2, we identified significant differences in abundances of several taxa between pre-transplantation and 1-week samples, as well as between pre-transplantation samples and those obtained 3 to 6 weeks post-transplantation (Fig. 2d, e). Samples were restricted, in this analysis, to taxa with at least 0.01% relative abundance in at least half of the samples for a given test combination, in order to ensure statistical power.

The most abundant species for which we observed significant changes was *Faecalibacterium prausnitzii.* Here, we observed a near twofold increase in the relative abundance in 1-week post-transplantation samples compared with samples taken pre-transplantation, and a 2.6-fold increase in the samples taken 3 to 6 weeks post-transplantation compared to pre-transplantation levels (*p* = 0.013 and *p* = 0.007, respectively). The relative abundance of *Akkermansia muciniphila* was reduced at both time points when compared with pre-transplantation samples (from 2.52% at pre-transplantation to 0.14% at 1 week post-transplantation, and further reduced to 0.02% at 3 to 6 weeks post-transplantation; *p* = 0.002 and *p* < 0.001, respectively). *A. muciniphila* was also significantly reduced at the 1-year visit relative to pre-transplantation (*p* < 0.009, Supplementary Fig. S6). There was an increase of *F. prausnitzii* from 4.2% pre-transplantation to 7% 1 year post-transplantation, but this difference was not statistically significant (*p* = 0.49).

There were other taxon differences between the groups. SGB1699 (family *Prevotellaceae*), SGB15356, SGB15106, and SGB15031 (all three assigned at the family level to *Ruminococcaceae*) also had lower relative abundances in the 1-week samples compared with pre-transplantation samples. While, *Roseburia intestinalis* and *Clostridium* sp. AF34_10BH showed significantly increased abundances at both 1 week and 3 to 6 weeks post-transplantation, compared to pre-transplantation levels (*p* < 0.01 for all comparisons, Fig. 2d, e).

### MPA reactivation rates correlate with β-GUS gene variants linked with *Faecalibacterium prausnitzii*

**Fig. 3:**
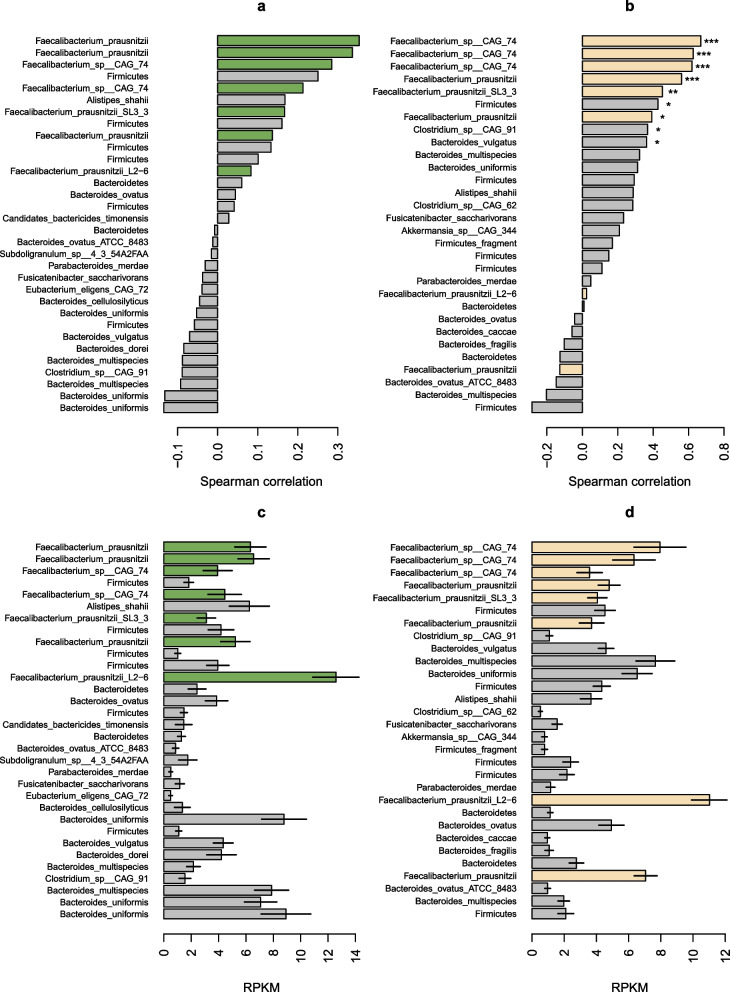
MPA reactivation rates correlate with β-GUSgene variants linked with *Faecalibacterium prausnitzii*. Correlations between MPA reactivation rates and read mapping rates to beta-glucuronidase (β-GUS) gene variants filtered to represent only those variants observed in at least 50% of kidney transplant recipients (**a**) and healthy individuals (**b**), as well as mean normalized abundances of those genes with error bars representing ± 1 s.e. (**c**,** d**). Green and beige bars represent β-GUS genes linked with *Faecalibacterium prausnitzii* in a/c and b/d, respectively. See Supplementary Fig. S7 for a more detailed label with the specific gene markers and Supplementary Fig. S8a and b for correlations between MPA reactivation rates and all observed β-GUS gene variants. *p < 0.05; **p < 0.01; ***p < 0.001. RPKM, reads per kilobase of target sequence per million reads in library

We then investigated if there were links between MPA reactivation rates and the prevalence of specific β-GUS genes. An assembly-free analysis of the β-GUS content in the metagenomes was done by mapping DNA sequence reads against a database containing 279 unique β-GUS gene sequences [25]. Notably, even though there was a higher reactivation rate in kidney transplant recipients post-transplantation vs. healthy individuals, the overall mapping rate to β-GUS genes (RPKM values) were not different between the two groups (Welch’s two sample *t*-test, *p* = 0.67). Correlations between the abundance of specific gene markers and the reactivation rates in both the kidney transplant recipients and the healthy individuals uncover that the largest correlations were with β-GUS markers found in *F. prausnitzii* (Fig. 3a, b, and Supplementary Fig. S7 and S8). Furthermore, five markers of high positive correlation were shared between the two groups, (such as SRS014923_34218-NL and SRS015663 18,708-NL), providing further evidence of the importance of this taxon in the reactivation of MPA from MPAG. In kidney transplant recipients, we also observed a negative correlation between the MPA reactivation rate and β-GUS gene variants found in *Bacteroides fragilis* (Supplementary Fig. S8a).

Interestingly, when comparing normalized mapping rates (RPKM; read per kilobase per million mapped reads), we found that there were high numbers of reads mapped to β-GUS genes, including some genes assigned to *F. prausnitzii* (such as SRS015264_139063-NL_Faecalibacterium_prausnitzii_L2.6) that were not highly correlated with the reactivation rates (Fig. 3c, d). This could signify that many β-GUS genes can be highly prevalent or abundant in a microbiome without being important for reactivating MPAG to MPA.

### Sequence comparison of β-GUS sequences assigned to Faecalibacterium prausnitzii

Since there was a range in the strength of correlations between the reactivation rate of MPA and the abundance of β-GUS gene variants found in *F. prausnitzii*, we compared the amino acid sequences of the 14 genes associated with this taxon to identify features that may indicate a higher or lower effectiveness at reactivating MPA (Supplementary Fig. S9a). The neighbor-joining tree of the full-length β-GUS sequences did not produce any obvious clustering that would differentiate highly correlated alleles with alleles that are not highly correlated with the reactivation rate.

Since previous studies have identified the catalytic domain of the protein that is important for the conversion of MPAG to MPA, we then extracted sequences of the catalytic sites of each enzyme, the so-called TIM barrel domain. This domain is found in all 14 markers, and we created a neighbor-joining tree from the alignment of these amino acid sequences. As we observed with the tree generated from the alignment of full-length sequences, the tree based on the TIM barrel sequences did not uncover an obviously discernible pattern. In fact, the top scoring hits for both datasets were separated by deep branches (Supplementary Fig. S9b). These findings highlight the need for further investigation to fully understand the functional implications of β-GUS and their role in MPA reactivation.

Recently, FMN-binding glucoronidases were identified in the human gut microboime [27]. In order to determine if the 14 *F. prausnitzii* β-GUSs described above can be considered FMN-binding enzymes, bioinformatics and structural analyses were performed. Multiple sequence alignments were generated using a reference *F. prausnitzii* FMN-binding β-GUS characterized by Pellock et al. (PDBID:6MVF) [27] (Supplementary Fig. S10). For further inspection of the putative FMN-binding sites in the 14 β-GUS enzymes, AlphaFold (AF) 3 models of all enzymes were aligned to the *F. prausnitzii* FMN-binding β-GUS (PDBID:6MVF). Based on the findings from the structure comparisons, as well as bioinformatics analyses, the 14 *F. prausnitzii* β-GUS enzymes in this study were categorized into four groups (Supplementary Fig. S11a), depending on the architecture and amino acid composition of the putative FMN-binding groove. Again, with the top scoring hits for both datasets being separated by deep branches (Supplementary Fig. S10 and S11). Based on our findings, we consider SRS015264_38260-NL_Faecalibacterium_prausnitzii_L2-6, SRS015663_18708-NL_Faecalibacterium_prausnitzii, and SRS015065_66511-NL_Faecalibacterium_prausnitzii to be FMN-binding proteins.

## Discussion

Our data demonstrate a substantial correlation between individual ex vivo determined MPA reactivation rates and the degree of enterohepatic recirculation in vivo, emphasizing the pivotal role of the gut microbiome in drug disposition. Utilizing metagenomics, we identified a significant association between specific β-GUS gene variants associated with *F. prausnitzii*, one of the predominant bacterial species of the human gut, and MPA reactivation. Additionally, our findings confirmed that kidney transplantation led to substantial alterations in microbial composition in the recipients. We specifically observed notable differences in the abundances of *F. prausnitzii* and *A. muciniphila* over various post-transplantation time points.

We collected fecal samples from kidney transplant recipients both before and after transplantation, as well as from healthy individuals. In transplant recipients, we observed a peak in MPA reactivation rates at 3 to 6 weeks post-transplantation, which returned to pre-transplantation levels after 1 year. This peak activity may be attributed to concurrent drugs used early post-transplant, including oral sulfamethoxazole/trimethoprim prophylaxis for 6 months post-transplantation, along with proton pump inhibitors and higher doses of prednisolone [8, 28, 29]. The reactivation rates in healthy individuals were consistent with pre-transplantation levels and those recorded at 1 week post-transplantation, but were lower than the rates observed at 3 to 6 weeks post-transplantation. We found significant interindividual variability in reactivation rates, with 14-fold and 23-fold differences observed in kidney transplant recipients and healthy individuals, respectively. Simpson et al. also examined the reactivation of MPA in similar populations and reported a considerably higher variability in reactivation rates among kidney transplant recipients compared to healthy individuals [26]. However, their methodology differed, as they determined MPA reactivation by assessing the depletion rate of MPAG, which makes direct comparisons to our results challenging. Additionally, the study by Simpson et al. had a limited sample size (five transplant recipients and four healthy individuals), further complicating the interpretation of whether the observed higher variability in patients reflects natural variability among transplant recipients, or if the findings are artefacts of the small sample size. Moreover, no MPA plasma concentrations were reported in their study, further limiting the comparison.

Importantly, we observed a strong correlation between the individual MPA reactivation rates and the degree of enterohepatic recirculation in kidney transplant recipients. This link between the reactivation rates and the in vivo pharmacokinetics of MPA within the same individuals is a novel finding of our study. Previous studies have explored associations between general β-GUS activity and/or microbiome composition with MPA pharmacokinetics or toxicity [30–33]. For instance, a study on hematopoietic cell transplantation recipients compared the GI microbiome between groups with high and low enterohepatic recirculation finding that the high enterohepatic recirculation group had elevated levels of *Bacteroides vulgatus*, *Bacteroides stercoris*, and *Bacteroides thetaiotaomicron* (30). Although *Bacteroides* is a well-known β-GUS producing genus, we did not observe a strong association between MPA reactivation and β-GUS gene variants linked to *Bacteroides* overall. Interestingly, our results showed a negative correlation specifically between MPA reactivation and β-GUS gene variants associated with *Bacteroides fragilis*. This correlation was only seen in the kidney transplant group, and the gene variants were present in fewer than 50% of the samples. Because of the limited number of samples with these variants and the fact that the finding was specific to one group, this should be interpreted with caution, and further studies are needed to confirm this observation. Taylor et al. measured β-GUS activity in human fecal samples from heart transplant recipients by using a commercial assay kit (hydrolysis of phenolphthalein glucuronide) and reported variable β-GUS activity that correlated with MMF-related side effects [31]. We demonstrated a moderate correlation between general β-GUS activity, as measured by a commercial kit, and the degree of enterohepatic recirculation. This suggest that general β-GUS activity is insufficient to fully explain the reactivation of MPA from MPAG, further supporting the notion of substrate specificity for β-GUS enzymes.

Since we observed strong correlations between the MPA reactivation rate and the degree of in vivo enterohepatic recirculation, we characterized the GI microbial community of the kidney transplant recipients and the healthy individuals using DNA shotgun metagenomics. As we, and others, have reported, the strongest structuring factor is the individual [34]. Still, significant differences were observed between the kidney transplant recipients and healthy individuals, with a significant increase in Bray–Curtis distance in the transplant recipients, regardless of the time of sampling, suggesting that increased β-diversity may be a general feature in this patient population, both before and after transplantation. This phenomenon has been observed in a number of dysbiotic conditions and has been proposed as an Anna Karenina principle for microbiomes, i.e., dysbiosis does not necessarily stem from specific alterations to the microbiome, but rather from increased instability and stochastic dynamics as a result of health issues [35–37]. Interestingly, we did not see differences in α-diversity between healthy individuals and patients pre-transplant, indicating that decreased diversity is not an important factor in the apparent dysbiosis in the kidney transplant recipients, again in agreement with an Anna Karenina-type hypothesis. We did, however, see significant drops in diversity post-transplant, which is likely related to the concurrent use of antibiotics and immunosuppressive drugs. Antibiotics in specific are a well-known factor in promoting decreased α-diversity [38, 39]. A previous study of the relationship between kidney transplantation and the GI microbiome did not find any such effect, possibly due to the small sample size [26]. However, consistent with our findings, Fricke et al. reported a reduction in α-diversity following transplantation, with the most significant shift occurring in fecal samples taken before and 1 month after transplantation [40].

A handful of studies examining the GI microbiome before and/or after transplantation have produced inconsistent results [7, 40–43]. Our main observations are the significant increases in *F. prausnitzii* at 1 week and 3 to 6 weeks after transplantation compared to the pre-transplantation levels. Furthermore, we observed a notable decrease in the relative abundance of *A. muciniphila* across all post-transplantation time points, dropping to negligible levels compared to pre-transplantation samples. *A. muciniphila* is positively correlated with mucus layer thickness and integrity in humans [44] suggesting that its post-transplantation decrease may be linked to the MMF-related GI toxicity in kidney transplant recipients. Other studies have reported similar taxonomic shifts. Fricke et al. found an increase of *Escherichia coli*, *Bacteroides and Clostridium *[40] 6 months following transplantation. Lee et al. also noted an increase in *Firmicutes and Bacteroidetes* post-transplantation [43]. Conversely, Swarte et al. reported that more than 1-year post-transplantation, kidney transplant recipients had increased abundance of *Proteobacteria* and *Firmicutes*, but decreased levels of *Actinobacteria* and butyrate-producing bacteria such as *F. prausnitzii* [7]*.* The main limitation of these studies is their comparison of post-transplantation microbiome composition with either the baseline (pre-transplantation) condition or with healthy individuals, making it challenging to isolate the specific effects of kidney transplantation from changes caused by the immunosuppressive therapy. Additionally, varying methodologies, including differences in antibiotic prophylaxis protocols further complicate direct comparison across studies.

In addition to taxonomic profiling, we tried to link the complement of microbial β-GUS genes in the microbiome with the MPA reactivation rate. To this end we computed the correlation of the normalized abundance of 279 unique β-GUS genes, with the reactivation rate in both the kidney transplant recipients and healthy individuals. Interestingly, we did not observe an increase in the mapping rate to β-GUS genes in the patient group relative to the healthy, even though the reactivation rate was higher in the patients. This suggests that the amount of glucuronidated drug is not enough to significantly perturb the microbiome or that the substrate specificity of different glucuronidase variants is more important than the total amount of genes in the metagenome. It is important to recognize the limitation that variations in specific protein levels present in fecal samples were not accounted for, which could influence these observations. Since metagenomic analysis reveals only the metabolic potential of the bacterial community, assessing actual utilization would require additional proteomic or transcriptomic insights.

Previous work, using an in vitro approach, was unable to explain differences in MPA reactivation rates with specific structural classes of genes [26]. Using the correlation approach we saw some clear patterns that might implicate the role of specific β-GUS variants, and their harboring taxa, in MPA reactivation efficiency. Specifically, in the kidney transplant recipients, six out of the top nine gene variants with the highest correlation were found in *F. prausnitzii*, or unassigned Faecalibacterium species, while in the healthy individuals the corresponding result was six out of the top seven. While the order of strength of correlation differed between these sets of genes, all variants were shared between the two groups. This strongly implicates *F. prausnitzii*, through its β-GUS genes, to drive the MPA reactivation. However, we also saw highly abundant Faecalibacterium-associated β-GUS variants that did not show a high degree of correlation. This lends to the notion that certain gene variants are more important than others in MPA reactivation, as has been reported previously [26].

Comparative amino acid sequence analysis of full length Faecalibacterium-associated β-GUS gene variants failed to produce clustering that reflected the correlation result, i.e., the top correlating gene variants did not cluster together. This was also the case when comparing only the catalytic domain (TIM barrel), which was identified in all 14 Faecalibacterium-associated genes. In fact, 4 representative TIM barrel sequences that were highly correlated in both groups were separated by deep branches. Further analysis of the 14 *F. prausnitzii* β-GUS enzymes categorized them according to the architecture and amino acid composition of the putative FMN-binding groove. Again, with the top scoring hits for both datasets being separated by deep branches.

AUC is regarded as the most reliable metric for guiding MMF dosing [45]. However, determining AUC is cumbersome and requires extensive sampling over the dosing interval, making it challenging to implement in a clinical setting. Limited sampling strategies based on population pharmacokinetic models offer an alternative by using a fewer number of timed samples to estimate AUC. The highly variable and complex pharmacokinetics of MPA pose challenges in developing these models, especially due to the very variable secondary peak that appear late and at different time-points after dosing. Despite many recent advances, most models have limited predictive accuracy and are currently not suitable for clinical TDM [46]. Incorporating individual MPA reactivation rates into these models may enhance models ability to predict individual enterohepatic recirculation, leading to more accurate overall AUC estimations. Consequently, this could enable improved and personalized MMF dosing for kidney transplant recipients, which again would give clinical benefits with less unwanted side effects and reduced rejection rate.

In this study, as with most others in this field [7, 40–43], patients were on multiple drugs after transplantation. Thus, it is challenging to attribute changes in microbial taxa to either kidney transplantation, immunosuppressive therapy, antibiotics or other concomitant drugs [23]. To address this, future studies should include a subgroup of kidney transplant recipients scheduled for living donor transplantation, allowing for in depth pre-transplant investigations. Another limitation of our study is that the fecal lysates were prepared from human fecal samples, while MPA reabsorption primarily occurs in the distal small intestine and proximal colon [15, 47]. Our previous study demonstrated that the composition of the GI microbiome varies across different sections of the GI tract [48]. However, we also found that the relative abundance of *F. prausnitzii* in the rectum was comparable to the levels observed in the distal small intestines [48]. Although fecal samples have some limitations, they remain the best and least invasive matrix for studying the gut microbiome. It is important to recognize the sex imbalance in our study populations, with the expected predominance of males in the kidney transplant group compared to more females among the healthy individuals. This disparity may have influenced our findings, given evidence from studies in mice suggesting sex related differences in β-GUS activity, and also the central role of β-GUS enzymes in estrogens reactivation [20, 49, 50]. Our study, however, was not sufficiently powered to specifically address sex-related variations in the reactivation of MPA. Despite these limitations, our results highlight the necessity of understanding the intricate host-microbiome-drug interactions for accurate and personalized drug dosing. Notably, we have demonstrated a strong correlation between the ex vivo determined reactivation rate of MPA and the degree of enterohepatic recirculation. Further, metagenomic investigations emphasized the important role of *F. prausnitzii* in this process. Our study also revealed significant shifts in microbial composition post-transplantation, with notable fluctuations in taxa such as *F. prausnitzii* and *A. muciniphila* across different time points after transplantation. Collectively, our findings suggest that the gut microbiome significantly influences MPA disposition and could potentially address the largely unexplained variability in drug response to MMF following transplantation. These insights may lay the foundation for more precise dosing strategies of MMF in kidney transplant recipients.

## Methods

### Patients and study design

Kidney transplant recipients of living- or deceased donor grafts were included before transplantation. This was an exploratory, prospective, pilot study that did not influence the pharmacological treatment of the patients. Fecal samples were collected prior to transplantation, at 1 week, between 3 and 6 weeks, and 1 year after transplantation (Supplementary Fig. S1a). A subset of the patients underwent a 12-h pharmacokinetic investigation of MMF in pharmacokinetic steady-state conditions 3 to 6 weeks with some repeating the investigation 1 year after transplantation. Fecal samples associated with these pharmacokinetic investigations were collected within 24-h of the start of investigation. Fecal samples were collected using a toilet specimen sampling kit (Fisher Scientific) and were kept at refrigerator temperature until stored at – 80 °C within 24 h. The study received approval from the Norwegian Medicine Agency (EudraCT: 2019–002476-14) and the Regional Committee for Medical and Health Research Ethics (2019/255289/REK). It was conducted in alignment with the principles of the Declaration of Helsinki and adhered to the Good Clinical Practice guidelines. Written informed consent was obtained from all participants prior to study inclusion.

Fecal samples from healthy individuals were collected in a previously conducted and published clinical study as outlined in Supplementary Fig. S1b (IntraCYP, 2021/255289/REK) [51]. This study was an open, prospective, single-armed, and single-center study also performed at Oslo University Hospital, Rikshospitalet, Norway. Healthy individuals with no underlying disease, aged 18 years or above, and with body mass index (BMI) < 30 kg/m^2^ were eligible for inclusion in the study. The participants were recruited from the Greater Oslo Region.

### Immunosuppression

Included transplant recipients were all standard immunological risk patients and received immunosuppressive therapy according to the protocol of our center. Induction with basiliximab 20 mg on days 0 and 4 post-transplantation, along with intravenous methylprednisolone 250 mg on day 0. Maintenance therapy consisted of a combination of tacrolimus, MMF and steroids. MMF was administered at a fixed dose of 750 mg twice daily from the day of transplantation Tacrolimus was initiated on the day of transplantation, administered as a starting dose of 0.04 mg/kg and adjusted to maintain a trough concentration (C_0_) between 4 and 7 μg/L. Prednisolone was administered according to a fixed tapering dose regimen, beginning at 20 mg/day the day after transplantation and tapered to a maintenance dose of 5 mg/day by 3 months.

### Pharmacokinetic investigation

On the pharmacokinetic investigation days, the participants fasted at least 10 h prior to the pharmacokinetic investigations except for water. Tacrolimus, MMF and prednisolone were administered simultaneously, with other concomitant drugs administered 2 h later. Blood samples for determination of plasma MPA concentrations were collected before (0 h) and 0.25, 0.5, 1, 1.5, 2, 3, 4, 5, 6, 7, 8, 9, 10, 11, and 12 h after morning administration of oral MMF. The precise sample times were recorded. Blood samples were drawn in K_2_-EDTA vacutainer tubes (2 mL Vacuette K_2_EDTA: Greiner Bio-One, Monroe, NC, USA) and centrifuged for 10 min at 4 ºC (1800 × *g*). Plasma was separated into Cryovials and stored at − 80 ºC until analysis.

### Fecal lysate preparation

Fecal lysates were prepared from human fecal samples, including those from kidney transplant recipients and healthy individuals using a modified version of the method introduced by Simpson et al. [26]. We have adapted and refined this method to better align with our experimental design. One gram of thawed fecal material from each patient was resuspended in 25 mL cold extraction buffer together with 500 mg autoclaved garnet beads and vortexed until homogenous (Vortex-Genie 2, Scientific Industries Inc, New York, USA). The extraction buffer contains 25 mM HEPES pH 6.5, 25 mM NaCl and one Roche Complete EDTA-free protease inhibitor tablet in 50 mL buffer. The samples were centrifuged at 500 g for 5 min at 4 °C, and the supernatant was collected. The remaining centrifuged pellet underwent an additional round of resuspension with 25 mL cold extraction buffer, and the vortex and centrifugation steps were repeated. Both supernatants were then combined and centrifuged at 500 × *g* for 10 min at 4 °C two additional times to remove insoluble fiber. The supernatant was then sonicated on a Fischer Scientific Sonic Dismembrator Model 500 four times with 20 s pulses. The lysate was then centrifuged at 17,000 × *g* for 30 min at 4 °C in a Beckman Coulter J2-HC centrifuge before it was passed through a 0.45 µm filter to remove insoluble debris. To remove small molecules and metabolites, the decanted lysate was concentrated with Amicon Ultra 15 mL 30 kDa centrifugal filters and washed with fresh extraction buffer. The total protein concentration of the final fecal lysate was quantified using a Bradford assay [52]. The fecal lysate was diluted to a total protein concentration of 200 mg/mL, aliquoted at 1 mL and then snap-frozen in liquid nitrogen and stored at − 80 °C until later use in the fecal lysate reactivation experiments.

### Reactivation of MPA in human fecal lysate

Initial conditions of the reactivation assay were based on the methodology reported by Simpson et al. [26]. Following further investigation into incubation times and varying combinations of MPAG concentration and fecal lysate protein concentration, slight modifications were made to optimize the final method. The results of MPA reactivation in fecal lysates at both 4 and 24 h are presented in Supplementary Fig. S12. Solid MPAG (Toronto Research Chemicals, CAT# M83150) was solubilized in 100% methanol at 33.4 mM and stored at − 80 °C until further use. MPAG (33.4 mM) was further diluted to a working concentration of 2 mM. Thawed human fecal lysate (total protein concentration, 200 μg/mL) was diluted with extraction buffer to a total protein concentration of 56 μg/mL. The reaction was initiated by adding 300 μL MPAG (2 mM) to 2.7 mL fecal lysate (56 μg/mL). Subsequently, 100 µL human fecal lysate spiked with MPAG was aliquoted in triplicates to all the endpoints (0 min, 15 min, 30 min, 1 h, 1.5 h, 2 h) and incubated in a water bath at 37 °C. The final reaction conditions were an MPAG concentration of 200 μM and a total protein concentration of 50 μg/mL at a final volume of 100 μL. The reaction was quenched with an equivalent volume (100 µL) of acetonitrile at the designated time points. Finally, the samples were centrifugated at 1500 × *g* for 10 min, before MPA and MPAG concentrations were quantified in the supernatant with the validated ultra-high-performance liquid chromatography (UPLC-MS/MS) method described in the *Bioanalytical assay* section. MPA concentrations as a function of time were fitted to a linear regression model, with the slope representing the reactivation rate of MPA from MPAG in μM/h. The reactivation rate represents the concentration of MPA formed per unit time.

Negative controls with extraction buffer and MPAG (200 μM) were included to ensure that no spontaneous hydrolysis of the glucuronide occurred, while blank controls with the fecal lysate (total protein concentration, 50 mg/mL) were included to determine the baseline concentration of MPA and MPAG. If baseline concentrations exceeded the lower limit of quantification (0.25 mg/L), these values were subtracted from the MPA and MPAG concentrations measured at subsequent time points in the reactivation assay prior to performing the linear regression. Experiments with Anaerobic Cultivated Human Intestinal Microbiota (ACHIM) were carried out in the same manner as described for the fecal lysates to obtain a reference MPA reactivation rate for method validation and calibration with a standardized culture [53]. Positive controls with ACHIM (total protein concentration, 50 mg/mL) and MPAG (200 μM) were included in the analysis of the patient samples to define the between-series precision of the MPA reactivation rate determinations and to ensure the reproducibility of the method. The ACHIM positive control samples had a coefficient of variation < 7% (Supplementary Table S3). In order to further demonstrate the specificity of the measurement techniques, we inhibited the conversion of MPAG to MPA using amoxapine, a well-established β-GUS inhibitor (Supplementary Fig. S13).

### Bioanalytical assay

Concentrations of MPA and MPAG in plasma and fecal lysates were determined by a previously validated UPLC-MS/MS method [54]. The method was slightly adapted for the fecal lysate samples; in brief, the analytes were purified by protein precipitation with acetonitrile (95%) and methanol (5%) containing internal standards (100 ng/mL MPA-d3 and MPAG-d3), prior to being separated chromatographically on a C18-column (Acquity UPLC HSS T3 1.8 μm 2.1 mm × 50 mm, Waters, Milford, MA, USA) with a guard column (Acquity UPLC HSS T3 1.8 μm 2.1 mm × 5 mm guard column, Waters, Milford, MA) before MS/MS detection (Vanquish UPLC connected to an Altis triple quadrupole, Thermo-Fisher, Waltham, MA, USA). Calibrators and quality control samples were prepared in Ringer acetate and analyzed in each series. Nine calibrators in the range of 0.25 to 64 mg/L were applied to both MPA and MPAG. Ringer acetate was used as a matrix for the calibrators and quality control samples instead of blank fecal lysate. The cross-validation between the two matrices demonstrated satisfactory accuracy and precision. No detectable carryover was observed following injection of the highest calibrator level or internal standard of both MPA and MPAG in blank fecal lysate. Between-series and within-series performance of MPA and MPAG in fecal lysates were assessed with resulting coefficients of variation < 6% and < 5%, respectively. The mean accuracy ranged from 98 to 109% for MPA and 92 to 103% for MPAG. For the plasma concentrations, between-series and within-series performance of MPA and MPAG were assessed with resulting coefficients of variation < 8% and < 9%, respectively. The mean accuracy ranged from 96 to 108% for MPA and 90 to 102% for MPAG.

### Determination of β-GUS activity using a commercial kit

β-GUS activity in fecal lysates was measured using a fluorometric assay kit (ab234625, Abcam, Cambridge, United Kingdom) following the manufacturer's instructions. The provided substrate was cleaved into a fluorescent product in the presence of β-GUS enzymes. Briefly, 10 μL of the proprietary substrate was added to 30 μL of fecal lysate and 60 μL of β-GUS assay buffer. Fluorescence measurement (excitation wavelength 330 nm and emission wavelength 450 nm) was done on a microplate reader (Wallac Victor® Nivo™, Perkin Elmer, Boston, MA, USA) immediately after adding substrate for 0–60 min at 37 °C. All reactions were conducted in black 96-well plates with flat bottoms (Thermo Fisher Scientific). Each run included a negative control (no substrate), positive control and a standard (4-methylumbelliferone, 4-MU). β-GUS activity was expressed as nmol 4-MU min^−1^.

### Pharmacokinetic calculations

All the pharmacokinetic calculations (non-compartmental) were carried out using Excel. The area under the plasma concentration–time curve from time 0 to 12 (AUC_0–12_) was calculated using the trapezoidal rule. The degree of enterohepatic recirculation (%) was calculated as the ratio of MPA AUC_*n*−12_/MPA AUC_0–12_ × 100, where *n* represents the last sampling time point before onset of recirculation in each individual patient. The correlation between the MPA reactivation rate and the degree of enterohepatic recirculation was estimated using the Spearman correlation test.

### DNA extraction, metagenomic sequencing, and DNA sequence data processing

DNA was extracted from the fecal samples using the MagAttract PowerMicrobiome DNA KF kit (Qiagen). Shotgun sequencing libraries were prepared using a tagmentation protocol with library size selection and sequenced on an Illumina NovaSeq 6000 instrument using a half S4 flow cell and 150 PE mode [55].

Raw sequence reads were trimmed and filtered using Trimmomatic v0.39 [56]. Reads with a host origin were identified by alignment to the human genome sequence CRCh38.p13 using Bowtie2 v2.4.2 [57] and removed using SAMTools v1.11 [58]. Read-based taxonomic assignment was done using MetaPhlAN 4 Version 4.0.6 [59]. Assembly-free analysis of β-GUS content in metagenomes was done using ShortBRED v0.9.4 [60] and a database containing 279 unique β-GUS gene sequences [25]. Protein domain categorization was performed using Interproscan version 5.62–94.0. Amino acid sequence alignments using MUSCLE, and neighbor joining tree construction was done with MEGA v10.1.7 [61].

### Inspection of putative FMN-binding sites in β-GUS variants from *F. prausnitzii*

Multiple sequence alignments (MSAs) of the 14 *F. prausnitzii* β-GUSs sequences described below were performed with Clustal Omega [62] and phylogenetic tree analysis with average distances using the BLOSUM62 matrix were performed in JalView [63]. As a reference *F. prausnitzii* FMN-binding β-GUS, the sequence of the enzyme characterized by Pellock et al. (PDBID:6MVF) was included [27]. Moreover, for higher confidence, four additional β-GUS sequences were included in the MSA, namely from *Roseburia inulinivorans* (locus tag CUM79767), *Ruminococcus gnavus* (PDBID:6MVG), *Roseburia hominis* (PDBID:6MVH), and *Butyrivibrio solvens* (locus tag WP_027217001). For further inspection of the putative FMN-binding sites in the 14 β-GUS enzymes, structure prediction was performed with AF 3 and ab initio models were generated of all enzymes [64], and structural alignments were made with the *F. prausnitzii* FMN-binding β-GUS (PDBID:6MVF).

### Statistical analysis

All statistical analysis were carried out with R [65]. PERMANOVA and non-metric multidimensional scaling (nMDS) were carried out with the functions “adonis2” and “metaMDS” in the vegan 2.6–4 package. PERMANOVA tests were carried out to check for effects on species β-diversity of individual sample provenance, patient vs. control samples, MPA reactivation rate and patient pre-transplant samples vs. 1 week, 3 to 6 weeks or 1 year post-transplant samples. Correlation tests were done with the “cor.test” function in the stats package. Benjamini–Hochberg false discovery rate correction was done with the ‘p.adjust’ function with method set to “BH”. Differential taxon analysis was done with DESeq2 v1.42.1. In order to retain statistical power, this particular analysis was restricted to taxa that were observed a at least 0.01% relative abundance in at least half of the samples for a given test combination. For testing for time trends in the data we use the Spearman’s correlation test in order to be conservative and not make assumptions about the underlying data distribution, which is poorly defined. Of the 50 samples from healthy individuals that were sequenced, 3 were filtered out due to small library size (less than 2.9 million reads). Fifteen of these samples, representing one sample from each of the healthy individuals, were selected based on the highest library size and used for comparing Bray–Curtis distances and Shannon entropies with kidney transplant recipients and to investigate whether the microbiome composition in kidney transplant recipients increasingly resembles healthy individuals over time.

## Supplementary Information

Supplementary Material 1. Supplementary Figure S1. Outline of the sampling study design and sampling procedure for the a) kidney transplant recipients and b) healthy individuals. Created with BioRender.com. Supplementary Figure S2. Individual mycophenolic acid (MPA) plasma concentration-time profiles. A pharmacokinetic investigation was performed in 10 kidney transplant recipients 3-6 weeks post-transplantation. Five of these patients repeating the investigation at one year, resulting in a total of 15 pharmacokinetic profiles of MPA. All patients received MMF doses of 750 mg twice daily. Supplementary Figure S3. Correlation between MPA reactivation rates (mM/h) and a) mycophenolic acid (MPA) area under the plasma concentration-time curve from time 0 to 12 (AUC_0-12_) (mg·h/L) and b) MPA trough concentration (C_12_) (mg/L) estimated using the Spearman correlation test (*n* = 15). Supplementary Figure S4. Correlation between general β-glucuronidase activity (nmol/min) measured using a commercial assay kit and a) degree of enterohepatic recirculation (%) and b) MPA reactivation rate (mM/h) estimated using the Spearman correlation test (*n* = 15). Supplementary Figure S5. The microbiome composition in kidney transplant recipients increasingly resembles healthy individuals over time. The boxes indicate the Bray-Curtis distances between healthy individuals and patients at one week pre-transplantation (Pre Tx), and one week, 3-6 weeks and one year post-transplantation. The analysis was conducted using Wilcoxon rank sum models with Benjamini-Hochberg correction of p-values. *p<0.05. Supplementary Figure S6. Barplots illustrating statistically significant differences in taxa relative abundances between fecal samples obtained pre-transplantation (beige) and one year after transplantation (blue). The analysis was conducted using DESeq2 (Differential Expression Sequencing) and was restricted to taxa that were observed with relative abundances of at least 0.01% in at least half of the samples for a given test combination. **p<0.01, ***p<0.001. Supplementary Figure S7. Correlations between MPA reactivation rates and read mapping rates to beta-glucuronidase (β-GUS) gene variants filtered to represent only those variants observed in at least 50% of kidney transplant recipients (a) and healthy individuals (b). (c and d) mean normalized abundances of those genes with error bars representing ±1 s.e. Green and beige bars represent β-GUS genes linked with *Faecalibacterium prausnitzii *in a/c and b/d, respectively. RPKM, reads per kilobase of target sequence per million reads in library. In contrast to main figure 3, here, full gene designations are included along the y-axes. Supplementary Figure S8a. Correlation between mycophenolic acid (MPA) reactivates rates and specific beta-glucuronidase (b-GUS) genes in kidney transplant recipients, analyzed using RPKM values (Reads per kilobase per million mapped reads). The green and red bars represent b-GUS genes associated with *Faecalibacterium prausnitzii *and *Bacteroides fragilis, *respectively. Supplementary Figure S8b. Correlation between mycophenolic acid (MPA) reactivates rates and specific beta-glucuronidase (b-GUS) genes in healthy individuals, analyzed using RPKM values (Reads per kilobase per million mapped reads). The green and red bars represent b-GUS genes associated with *Faecalibacterium prausnitzii *and *Bacteroides fragilis, *respectively. Supplementary Figure S9. Neighbor-joining trees of a) full-length beta-glucuronidase (b-GUS) sequences and b) Tim barrel sequences (the catalytic domain essential for MPAG to MPA conversion) in all 14 gene markers associated with *Faecalibacterium prausnitzii*. The five b-GUS markers of high positive correlation shared between kidney transplant recipients and healthy individuals have been highlighted in green boxes. No clear clustering was observed to differentiate highly correlated alleles with alleles that are not highly correlated with the reactivation of MPA. Supplementary Figure S10. Multiple sequence alignment and phylogenetic tree analysis of full-length b-GUS sequences from *F. prausnitzii* and four additional b-GUSs from selected *Bacillota*, with respect to putative FMN-binding. (a) Multiple sequence alignment generated with Clustal Omega through Jalview, colored according to % identity. The sequences are grouped according to the phylogenetic tree in (b). Amino acid residues previously shown to make key contacts with FMN are indicated with red asterisks (1). (b) Phylogenetic tree calculated in Jalview with average distances using the BLOSUM62 matrix on the sequence alignment in (a). The sequences cluster in groups according to the structural features observed in the structural overlays and inspection of the putative FMN-binding sites in Supplementary Figure 11, and are colored correspondingly. b-GUS sequences from *R. inulinivorans*, *R. gnavus*, *R. hominis*, and *B. solvens* are depicted in grey and not further discussed. Supplementary Figure S11. Comparison of putative FMN-binding sites in *F. prausnitzii* b-GUS enzymes. (a) Overview of amino acids in the potential FMN-binding site of 14 *F. prausnitzii* b-GUSs, compared to the FMN-bound *F. prausnitzii* b-GUS (PDBID:6MVF), indicated in the MSA in Supplementary Figure S10, and confirmed through structural alignments in (b), (c), (d), and (e). Based on the amino acid composition in the putative FMN-binding sites, the 14 b-GUS sequences have been divided into four groups colored in green, yellow, orange, and red, with decreasing likelihood of FMN-binding, respectively. Structural alignments of *F. prausnitzii* b-GUS (PDBID:6MVF) and the 14 b-GUS AF3 models from this study, showing the (b) enzymes considered as FMN-bound, containing two aromatic residues known to form p-p stacking interactions with the flavin isoalloxazine ring (shades of green), (c) b-GUS enzymes less likely to bind FMN (shades of yellow), and (d) and (e) b-GUS enzymes not likely to bind FMN (shades of orange and red). The loop harbouring the FMN-stacking Y154 (PDBID:6MVF numbering), protruding from the C-terminal jellyroll-like b-sandwich domain into the FMN-binding groove, flanked by the latter domain, a second b-sandwich domain, and the core TIM-barrel fold, is missing in the b-GUS structural models in (e). The FMN cofactor and selected amino acid sidechains in its vicinity are represented as sticks and colored by atom type. Supplementary Figure S12. Reactivation of MPA from MPAG in human fecal lysates, with incubation times of 48 hours (a) and 4 hours (b). The final reaction conditions were: a) MPAG concentration of 50 mg/L and total protein concentration of 50 mg/mL and b) MPAG concentration of 100 mg/L and total protein concentration of 50 mg/mL. The experiments were performed using lysates prepared from fecal samples collected from two healthy individuals. Supplementary Figure S13. MPA lysate concentrations (mg/L) as a function of time. Visualizing the reactivation of MPA from MPAG in human fecal lysates with and without different concentrations (0.1, 0.5, 2 and 5 mg/mL) of amoxapine (known inhibitor of b-glucuronidase enzymes). Supplementary Table S1. Demographic data and patient characteristics of the kidney transplant recipients at the time of transplantation (*n *= 22). Supplementary Table S2. Overview of the drug classes used by the kidney transplant recipients (*n *= 22). Supplementary Table S3. MPA reactivation rates for ACHIM control samples (*n*=11). Lower and upper limit values are presented as mean values ± 20%.

## Data Availability

Sequence data that support the findings of this study have been deposited in the NCBI SRA repository with the primary accession code PRJNA1206184.
