# Supplementary material for: Microbiome-derived reactivation of mycophenolate explains variations in enterohepatic recirculation in kidney transplant recipients
Source: Microbiome. 2025 Jul 24;13:169. doi: 10.1186/s40168-025-02142-6 (PMC12291295; doi:10.1186/s40168-025-02142-6)
Supplement: Supplementary file 2 — Supplementary Material 1. Supplementary Figure S1. Outline of the sampling study design and sampling procedure for the a) kidney transplant recipients and b) healthy individuals. Created with BioRender.com. Supplementary Figure S2. Individual mycophenolic acid (MPA) plasma concentration-time profiles. A pharmacokinetic investigation was performed in 10 kidney transplant recipients 3-6 weeks post-transplantation. Five of these patients repeating the investigation at one year, resulting in a total of 15 pharmacokinetic profiles of MPA. All patients received MMF doses of 750 mg twice daily. Supplementary Figure S3. Correlation between MPA reactivation rates (mM/h) and a) mycophenolic acid (MPA) area under the plasma concentration-time curve from time 0 to 12 (AUC0-12) (mg·h/L) and b) MPA trough concentration (C12) (mg/L) estimated using the Spearman correlation test (n = 15). Supplementary Figure S4. Correlation between general β-glucuronidase activity (nmol/min) measured using a commercial assay kit and a) degree of enterohepatic recirculation (%) and b) MPA reactivation rate (mM/h) estimated using the Spearman correlation test (n = 15). Supplementary Figure S5. The microbiome composition in kidney transplant recipients increasingly resembles healthy individuals over time. The boxes indicate the Bray-Curtis distances between healthy individuals and patients at one week pre-transplantation (Pre Tx), and one week, 3-6 weeks and one year post-transplantation. The analysis was conducted using Wilcoxon rank sum models with Benjamini-Hochberg correction of p-values. *p<0.05. Supplementary Figure S6. Barplots illustrating statistically significant differences in taxa relative abundances between fecal samples obtained pre-transplantation (beige) and one year after transplantation (blue). The analysis was conducted using DESeq2 (Differential Expression Sequencing) and was restricted to taxa that were observed with relative abundances of at least 0.01% in at least half of the s [file 40168_2025_2142_MOESM1_ESM.docx]

**SUPPLEMENTARY DIGITAL CONTENT**

**Microbiome-derived reactivation of mycophenolate explains variations in enterohepatic recirculation in kidney transplant recipients**

Ole Martin Drevland MSc^1^, Eric J. de Muinck PhD^1,2^, Pål Trosvik PhD^2^, Marta Hammerstad PhD^3^, Kine Eide Kvitne PhD^1,4^, Karsten Midtvedt MD, PhD^5^, Anders Åsberg PhD^1,5^, Ida Robertsen PhD^1^.

**Affiliations:**

*^1^Section for Pharmacology and Pharmaceutical Biosciences, Department of Pharmacy, University of Oslo, Oslo, Norway*

*^2^Centre for Ecological and Evolutionary Synthesis, Department of Biosciences, University of Oslo, Oslo, Norway*

*^3^Section for Biochemistry and Molecular Biology, Department of Biosciences, University of Oslo, Oslo, Norway*

*^4^Skaggs School of Pharmacy and Pharmaceutical Sciences, University of California San Diego, La Jolla, CA, USA*

*^5^Department of Transplantation Medicine, Oslo University Hospital, Oslo, Norway*

**Corresponding authors:**

Ole Martin Drevland
Department of Pharmacy, University of Oslo, P.O. Box 1068 Blindern, 0316 Oslo, Norway
E-mail: [oledre@farmasi.uio.no](mailto:oledre@farmasi.uio.no)

Ida Robertsen
Department of Pharmacy, University of Oslo, P.O. Box 1068 Blindern, 0316 Oslo, Norway
E-mail: [ida.robertsen@farmasi.uio.no](mailto:oledre@farmasi.uio.no)

**Supplementary Figures**

**
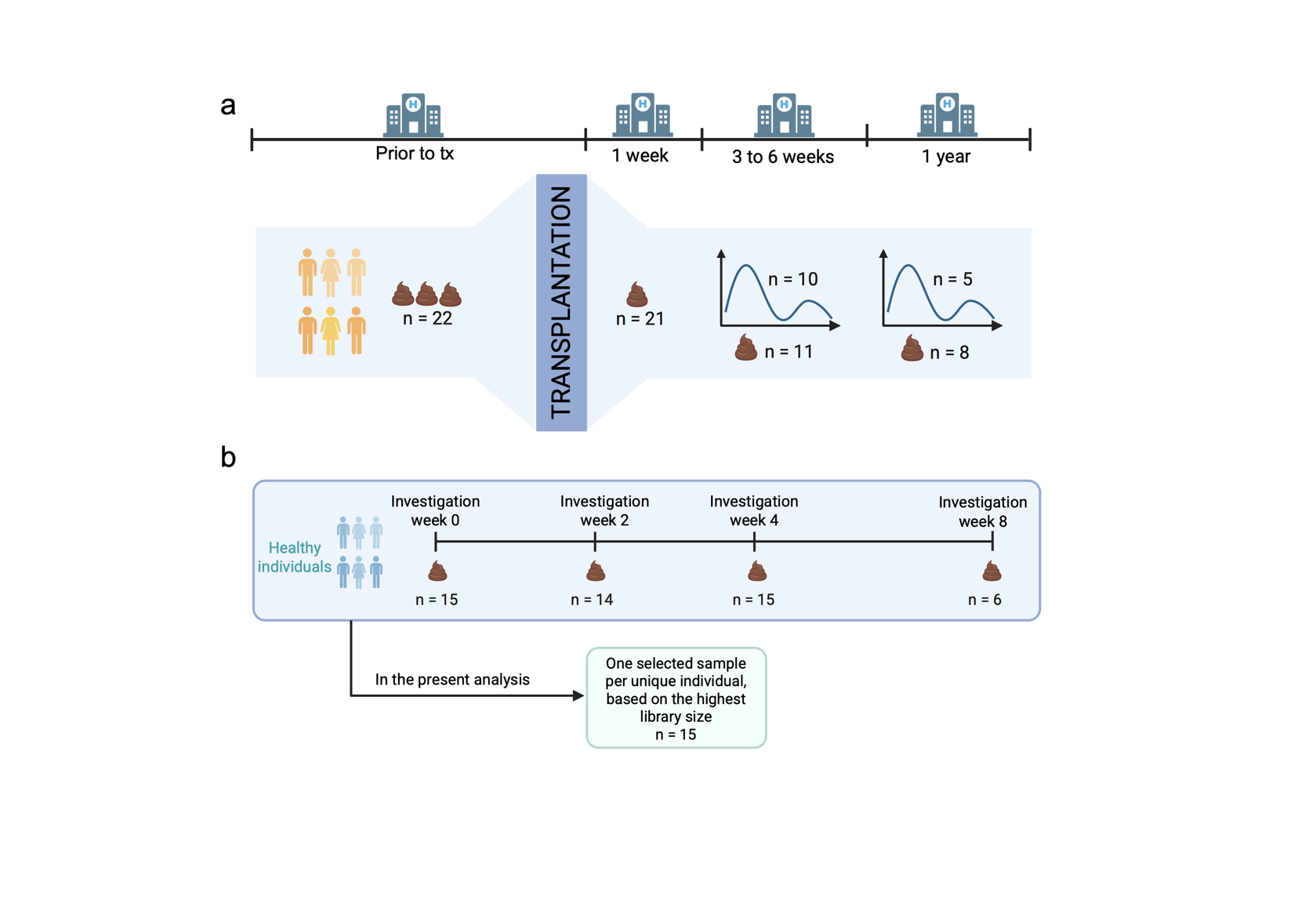
**

**Supplementary Figure 1:** Outline of the sampling study design and sampling procedure for the a) kidney transplant recipients and b) healthy individuals. Created with BioRender.com


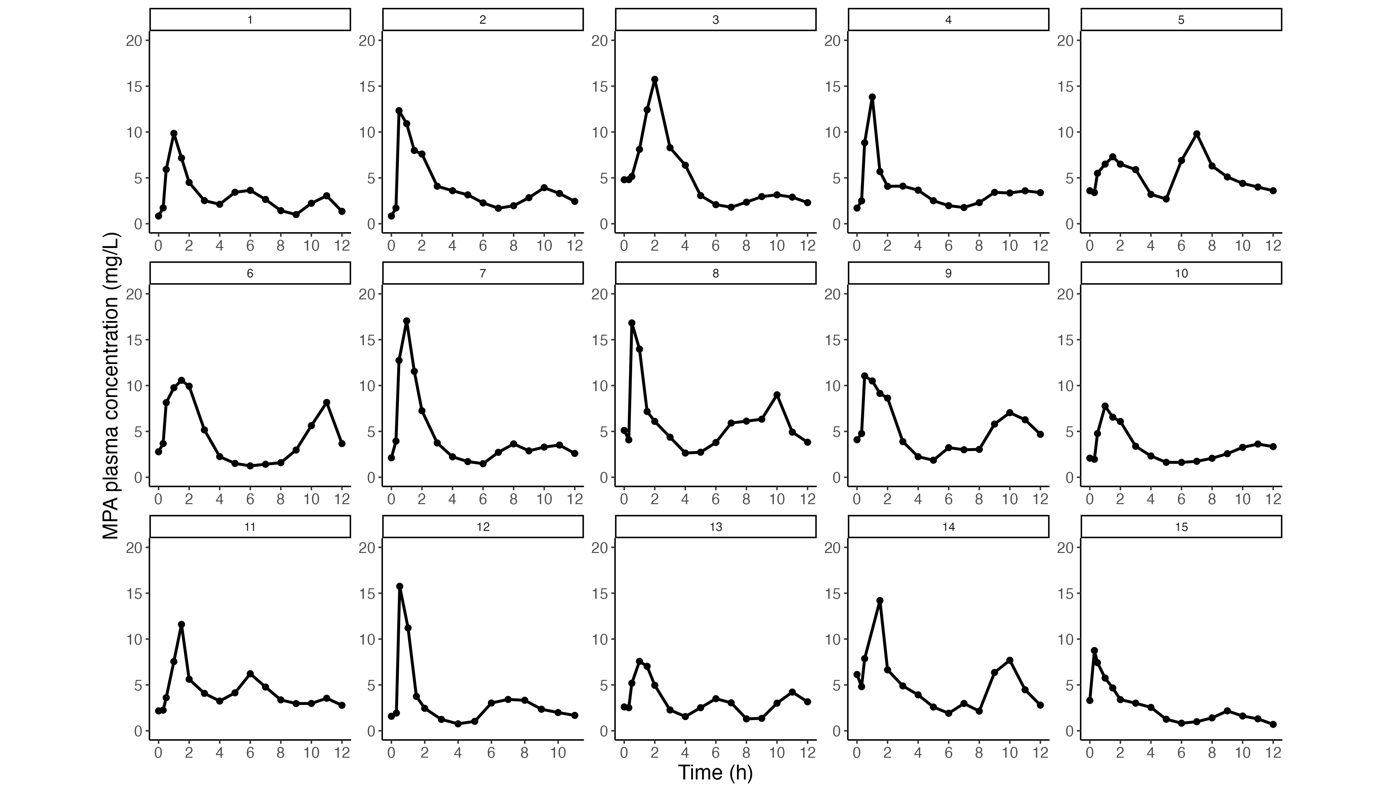


**Supplementary Figure 2:** Individual mycophenolic acid (MPA) plasma concentration-time profiles. A pharmacokinetic investigation was performed in 10 kidney transplant recipients 3-6 weeks post-transplantation. Five of these patients repeating the investigation at one year, resulting in a total of 15 pharmacokinetic profiles of MPA. All patients received MMF doses of 750mg twice daily.


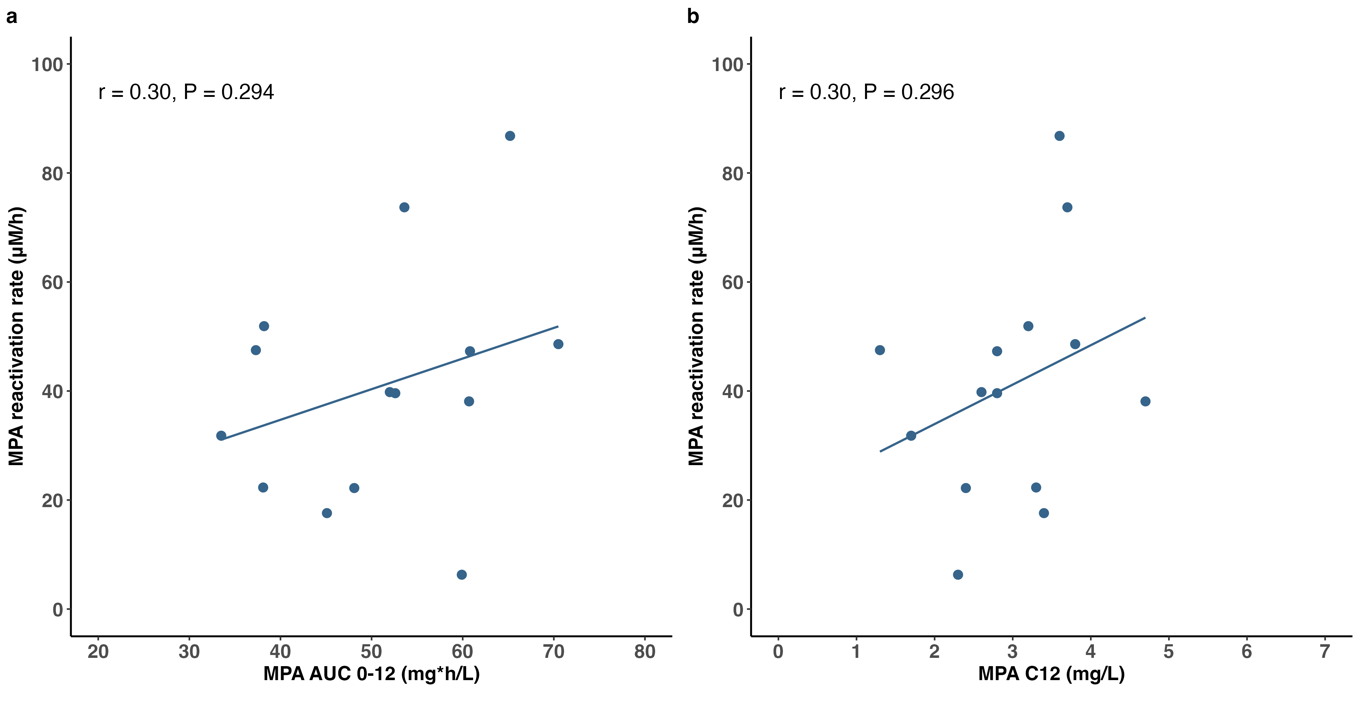


**Supplementary Figure 3:** Correlation between MPA reactivation rates (μM/h) and a) mycophenolic acid (MPA) area under the plasma concentration-time curve from time 0 to 12 (AUC_0-12_) (mg·h/L) and b) MPA trough concentration (C_12_) (mg/L) estimated using the Spearman correlation test (*n* = 15).

**
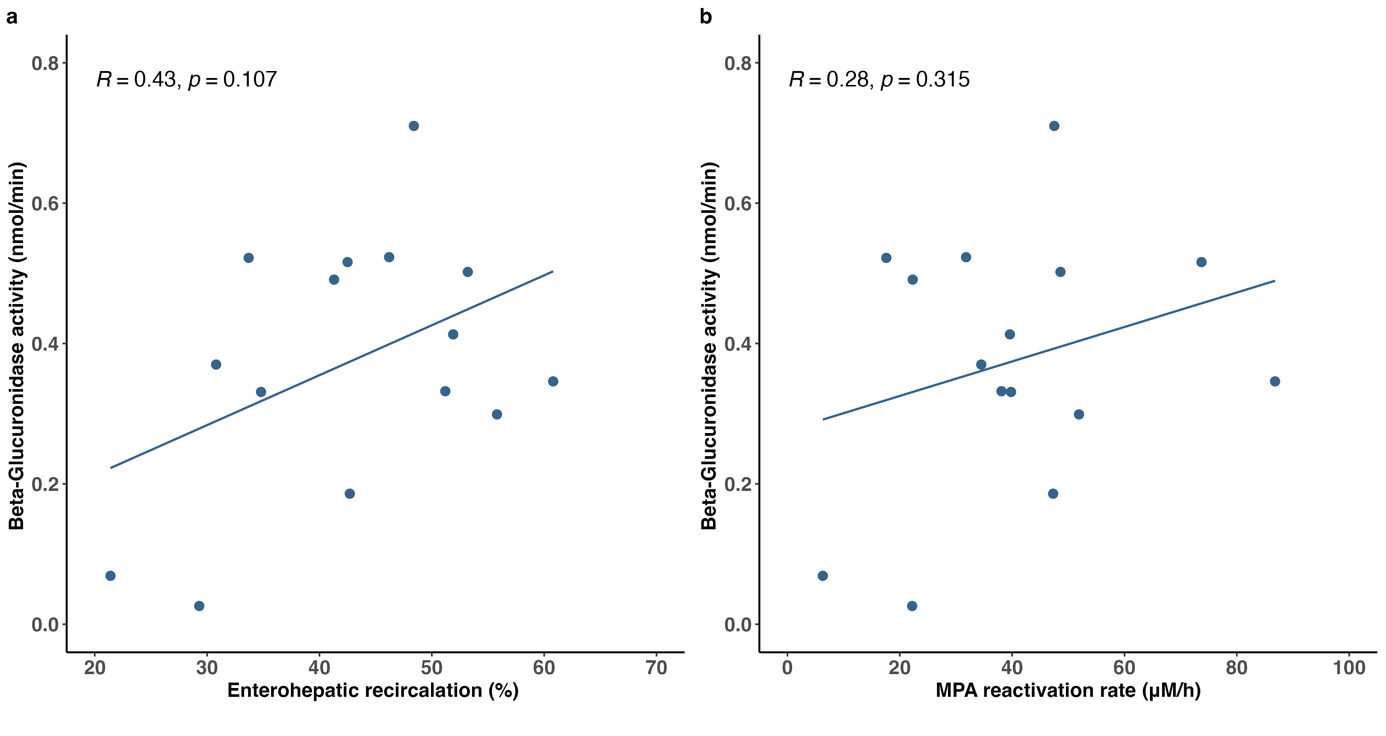
**

**Supplementary Figure 4:** Correlation between general β−glucuronidase activity (nmol/min) measured using a commercial assay kit and a) degree of enterohepatic recirculation (%) and b) MPA reactivation rate (μM/h) estimated using the Spearman correlation test (*n* = 15).

**Supplementary Figure 5:** The microbiome composition in kidney transplant recipients increasingly resembles healthy individuals over time. The boxes indicate the Bray-Curtis distances between healthy individuals and patients at one week pre-transplantation (Pre Tx), and one week, 3-6 weeks and one year post-transplantation. The analysis was conducted using Wilcoxon rank sum models with Benjamini-Hochberg correction of p-values. *p<0.05

**Supplementary Figure 6:** Barplots illustrating statistically significant differences in taxa relative abundances between fecal samples obtained pre-transplantation (beige) and one year after transplantation (blue). The analysis was conducted using DESeq2 (Differential Expression Sequencing) and was restricted to taxa that were observed with relative abundances of at least 0.01% in at least half of the samples for a given test combination. **p<0.01, ***p<0.001.

**Supplementary Figure 7:** Correlations between MPA reactivation rates and read mapping rates to beta-glucuronidase (β-GUS) gene variants filtered to represent only those variants observed in at least 50% of kidney transplant recipients **(a)** and healthy individuals **(b)**. **(c and d)** mean normalized abundances of those genes with error bars representing ±1 s.e. Green and beige bars represent β-GUS genes linked with *Faecalibacterium prausnitzii* in **a/c** and **b/d**, respectively. RPKM, reads per kilobase of target sequence per million reads in library. In contrast to main figure 3, here, full gene designations are included along the y-axes.

**Supplementary Figure 8a:** Correlation between mycophenolic acid (MPA) reactivates rates and specific beta-glucuronidase (β-GUS) genes in kidney transplant recipients, analyzed using RPKM values (Reads per kilobase per million mapped reads). The green and red bars represent β-GUS genes associated with *Faecalibacterium prausnitzii* and *Bacteroides fragilis,* respectively.

**

**Supplementary Figure 8b:** Correlation between mycophenolic acid (MPA) reactivates rates and specific beta-glucuronidase (β-GUS) genes in healthy individuals, analyzed using RPKM values (Reads per kilobase per million mapped reads). The green and red bars represent β-GUS genes associated with *Faecalibacterium prausnitzii* and *Bacteroides fragilis,* respectively.

*
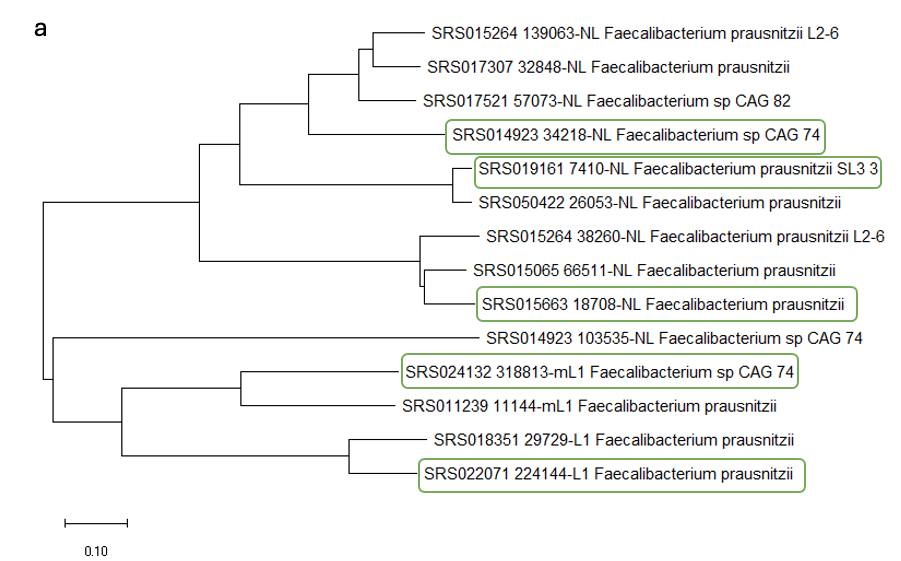
*


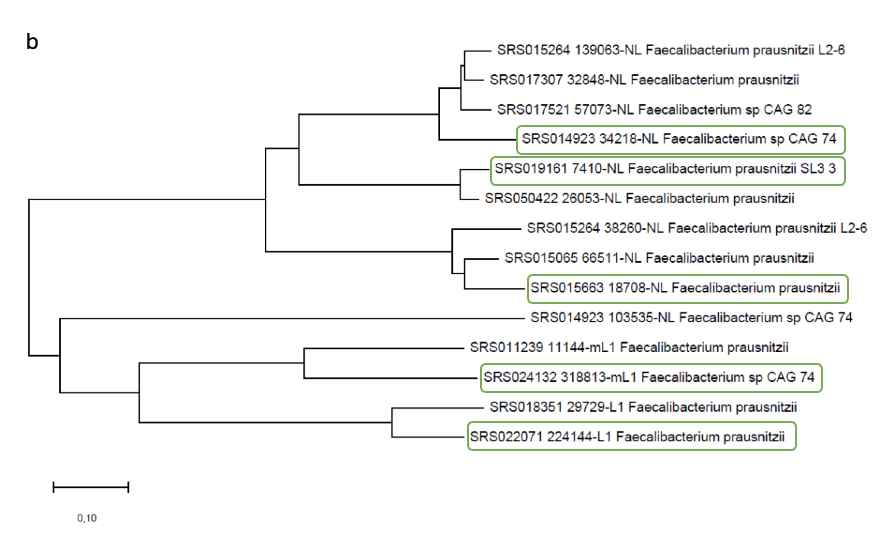


**Supplementary Figure 9:** Neighbor-joining trees of a) full-length beta-glucuronidase (β-GUS) sequences and b) Tim barrel sequences (the catalytic domain essential for MPAG to MPA conversion) in all 14 gene markers associated with *Faecalibacterium prausnitzii*. The five β-GUS markers of high positive correlation shared between kidney transplant recipients and healthy individuals have been highlighted in green boxes. No clear clustering was observed to differentiate highly correlated alleles with alleles that are not highly correlated with the reactivation of MPA.


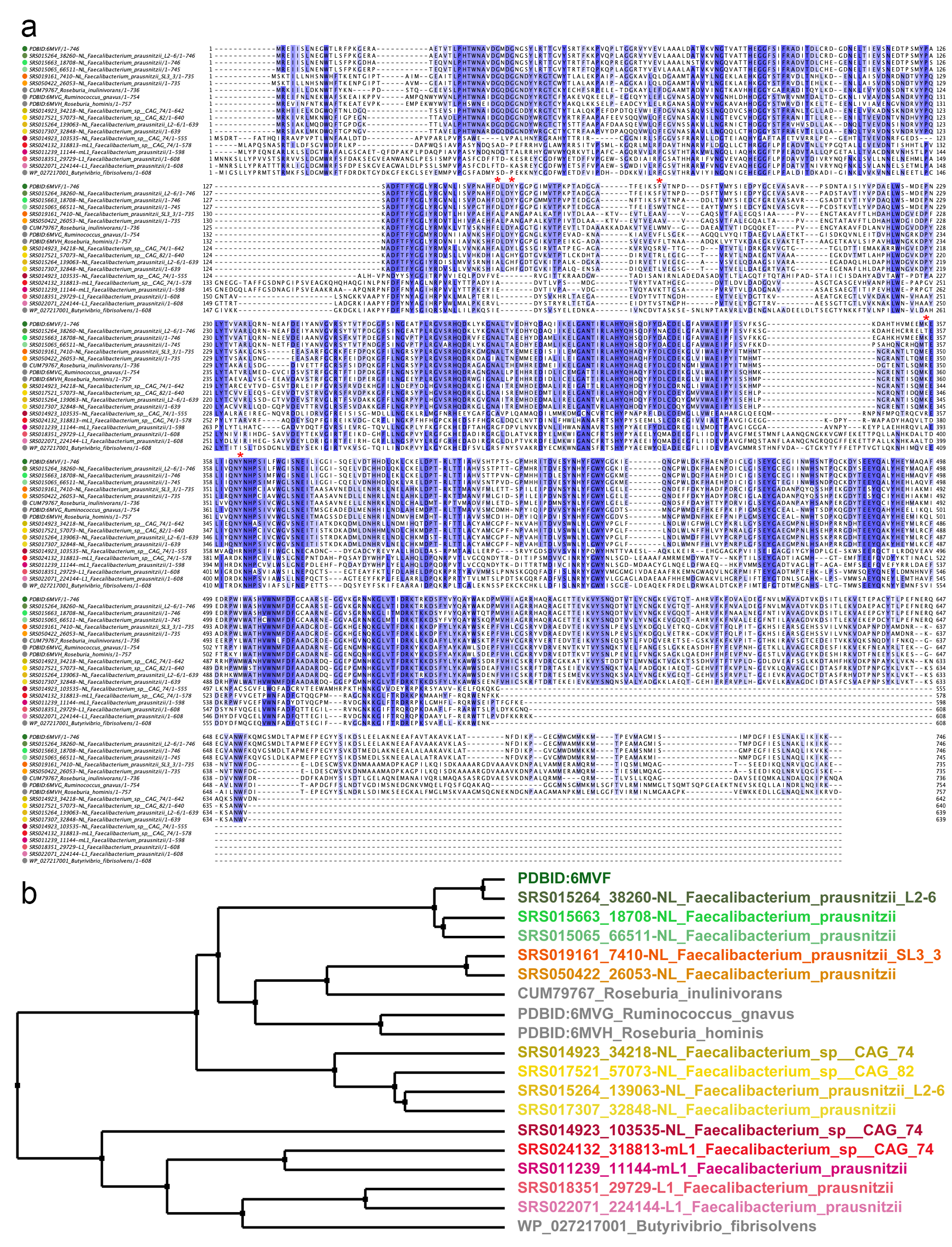


**Supplementary Figure 10:** Multiple sequence alignment and phylogenetic tree analysis of full-length β-GUS sequences from *F. prausnitzii* and four additional β-GUSs from selected *Bacillota*, with respect to putative FMN-binding. **(a)** Multiple sequence alignment generated with Clustal Omega through Jalview, colored according to % identity. The sequences are grouped according to the phylogenetic tree in (b). Amino acid residues previously shown to make key contacts with FMN are indicated with red asterisks (1). **(b)** Phylogenetic tree calculated in Jalview with average distances using the BLOSUM62 matrix on the sequence alignment in (a). The sequences cluster in groups according to the structural features observed in the structural overlays and inspection of the putative FMN-binding sites in Supplementary Figure 11, and are colored correspondingly. β-GUS sequences from *R. inulinivorans*, *R. gnavus*, *R. hominis*, and *B. solvens* are depicted in grey and not further discussed.


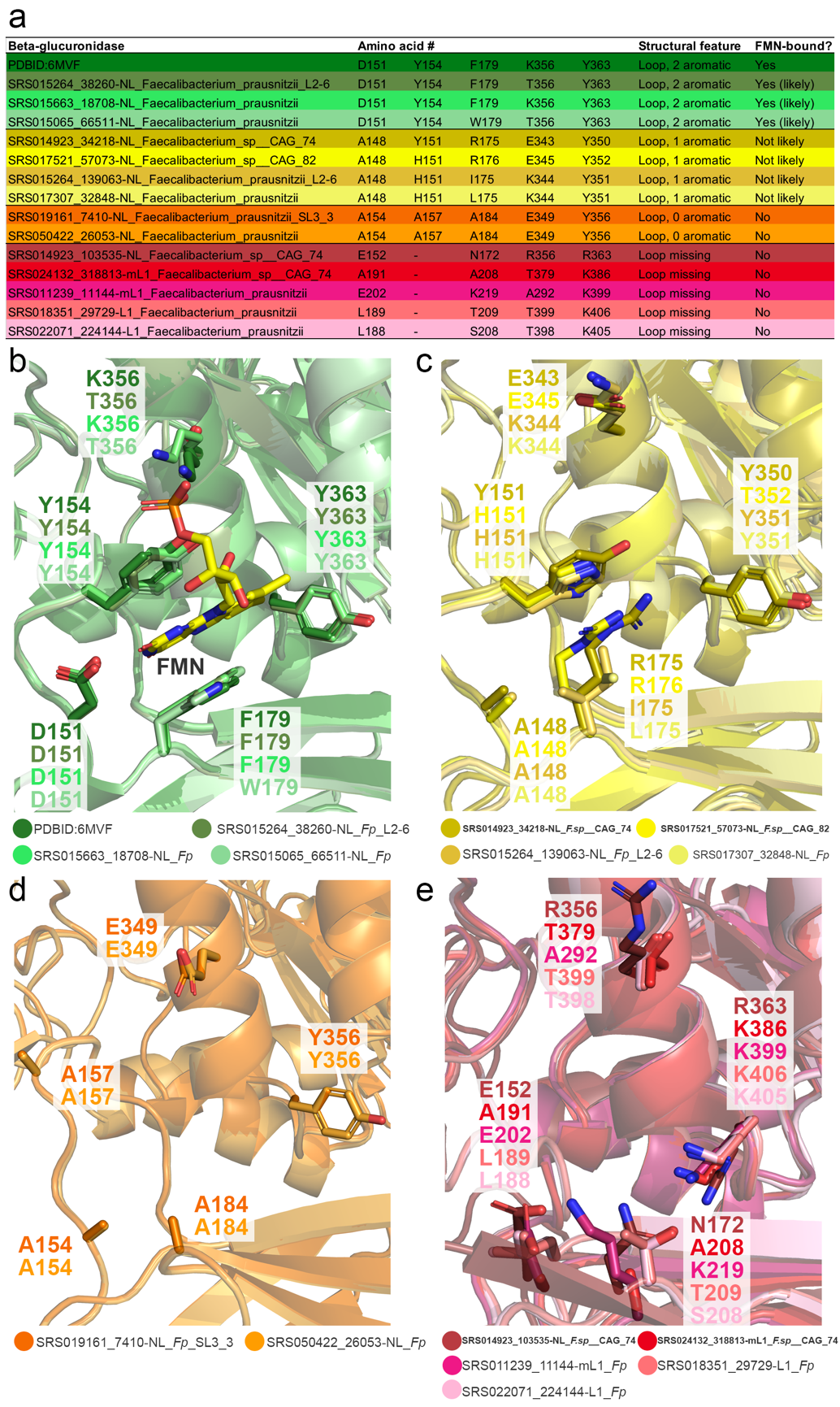


**Supplementary Figure 11:** Comparison of putative FMN-binding sites in *F. prausnitzii* β-GUS enzymes. **(a)** Overview of amino acids in the potential FMN-binding site of 14 *F. prausnitzii* β-GUSs, compared to the FMN-bound *F. prausnitzii* β-GUS (PDBID:6MVF), indicated in the MSA in Supplementary Figure 10, and confirmed through structural alignments in (b), (c), (d), and (e). Based on the amino acid composition in the putative FMN-binding sites, the 14 β-GUS sequences have been divided into four groups colored in green, yellow, orange, and red, with decreasing likelihood of FMN-binding, respectively. Structural alignments of *F. prausnitzii* β-GUS (PDBID:6MVF) and the 14 β-GUS AF3 models from this study, showing the **(b)** enzymes considered as FMN-bound, containing two aromatic residues known to form π-π stacking interactions with the flavin isoalloxazine ring (shades of green), **(c)** β-GUS enzymes less likely to bind FMN (shades of yellow), and **(d)** and **(e)** β-GUS enzymes not likely to bind FMN (shades of orange and red). The loop harbouring the FMN-stacking Y154 (PDBID:6MVF numbering), protruding from the C-terminal jellyroll-like β-sandwich domain into the FMN-binding groove, flanked by the latter domain, a second β-sandwich domain, and the core TIM-barrel fold, is missing in the β-GUS structural models in (e). The FMN cofactor and selected amino acid sidechains in its vicinity are represented as sticks and colored by atom type.


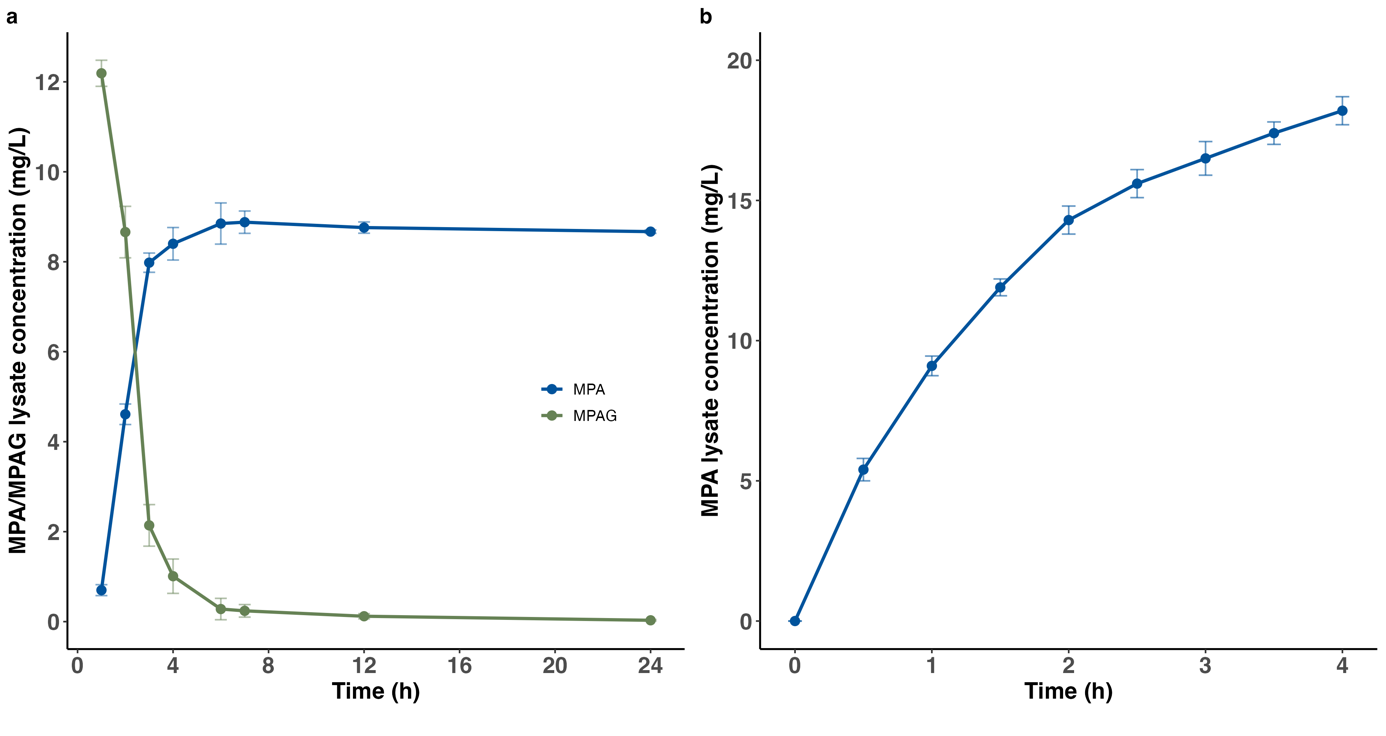


**Supplementary Figure 12:** Reactivation of MPA from MPAG in human fecal lysates, with incubation times of 48 hours (a) and 4 hours (b). The final reaction conditions were: a) MPAG concentration of 50 mg/L and total protein concentration of 50 μg/mL and b) MPAG concentration of 100 mg/L and total protein concentration of 50 μg/mL. The experiments were performed using lysates prepared from fecal samples collected from two healthy individuals.

**
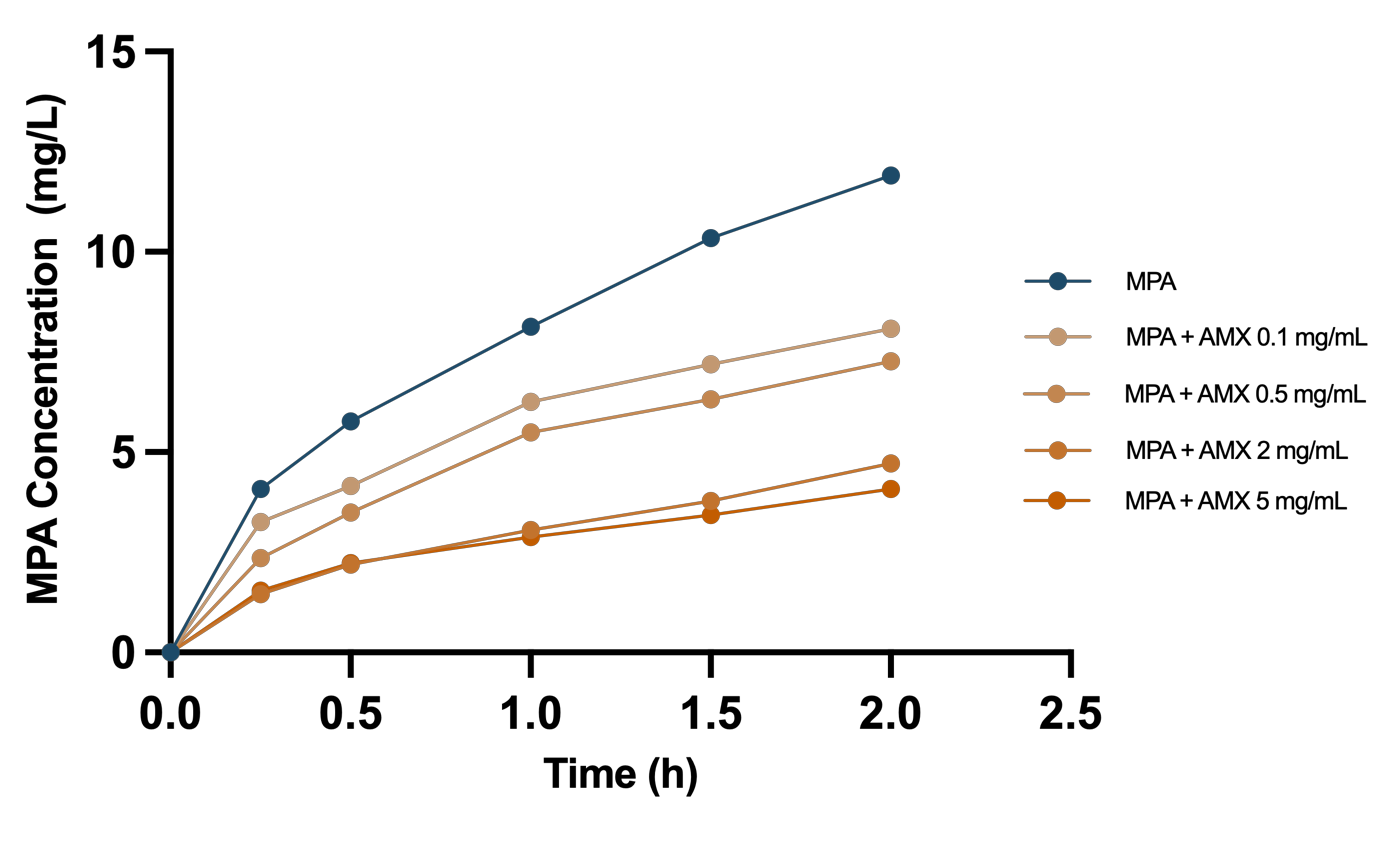
**

**Supplementary Figure 13:** MPA lysate concentrations (mg/L) as a function of time. Visualizing the reactivation of MPA from MPAG in human fecal lysates with and without different concentrations (0.1, 0.5, 2 and 5 mg/mL) of amoxapine (known inhibitor of β-glucuronidase enzymes).

**Supplementary Tables**

**Supplementary Table 1.** Demographic data and patient characteristics of the kidney transplant recipients at the time of transplantation (*n* = 22).

| Sex (male/female), *n* | 16/6 |
| --- | --- |
| Age (years) | 55 (28-77) |
| BMI (kg/m^2^) | 27 (20-35) |
| Donor (living/dead), *n* | 17/5 |
| Dialysis (yes/no), *n* | 11/11 |
| Time in dialysis (days) | 188 (54-1013) |
| Smoking, *n* |  |
| Smoker | 4 |
| Ex-smoker | 6 |
| Never smoked | 12 |

Data is presented as numbers or median (range). BMI, body mass index

**Supplementary Table 2.** Overview of the drug classes used by the kidney transplant recipients (*n* = 22).

| **Drug class** | ***n*** |
| --- | --- |
| Alpha_1_-blockers | 5 |
| Angiotensin II receptor blockers | 5 |
| Antihistamines | 2 |
| Antivirals | 3 |
| Beta-blockers | 8 |
| Calcium channel blockers | 11 |
| GLP-1 agonists | 4 |
| HMG-CoA reductase inhibitors | 5 |
| Loop diuretics | 7 |
| NSAIDs | 8 |
| Opioid analgesics | 2 |
| Proton-pump inhibitors | 15 |
| SNRIs | 1 |
| Xanthine oxidase inhibitors | 4 |

HMG CoA, Hydroxy-Methylglutaryl Coenzyme A; GLP-1, Glucagon-like Peptide-1; NSAIDs, Nonsteroidal Anti-inflammatory Drugs; SNRIs, Serotonin and Norepinephrine Reuptake Inhibitors

**Supplementary Table 3.** MPA reactivation rates for ACHIM control samples (*n*=11). Lower and upper limit values are presented as mean values ± 20%.

| **Mean ± SD**  **(µM/h**) | **Imprecision**  **(CV, %)** | **Lower limit**  **(µM/h**) | **Upper limit**  **(µM/h**) |
| --- | --- | --- | --- |
| 1.3 ± 0.1 | 6.6 | 1.04 | 1.56 |

ACHIM, Anaerobic Cultivated Human Intestinal Microbiota; SD, Standard deviation; CV, Coefficient of variation.

**References**

1. Pellock SJ, Walton WG, Ervin SM, Torres-Rivera D, Creekmore BC, Bergan G, et al. Discovery and Characterization of FMN-Binding β-Glucuronidases in the Human Gut Microbiome. J Mol Biol. 2019;431(5):970-80.
